# Structural variants in the Chinese population and their impact on phenotypes, diseases and population adaptation

**DOI:** 10.1038/s41467-021-26856-x

**Published:** 2021-11-11

**Authors:** Zhikun Wu, Zehang Jiang, Tong Li, Chuanbo Xie, Liansheng Zhao, Jiaqi Yang, Shuai Ouyang, Yizhi Liu, Tao Li, Zhi Xie

**Affiliations:** 1 State Key Laboratory of Ophthalmology, Zhongshan Ophthalmic Center, Sun Yat-sen University, Guangzhou, China; 2 Sun Yat-sen University Cancer Center, Sun Yat-sen University, Guangzhou, China; 3 Mental Health Center and Psychiatric Laboratory, the State Key Laboratory of Biotherapy, West China Hospital of Sichuan University, Chengdu, China; 4 Guangdong-Hong Kong-Macao Greater Bay Area Center for Brain Science and Brain-Inspired Intelligence, Guangzhou, China

**Keywords:** Population genetics, Structural variation, Structural variation

## Abstract

A complete characterization of genetic variation is a fundamental goal of human genome research. Long-read sequencing has improved the sensitivity of structural variant discovery. Here, we conduct the long-read sequencing-based structural variant analysis for 405 unrelated Chinese individuals, with 68 phenotypic and clinical measurements. We discover a landscape of 132,312 nonredundant structural variants, of which 45.2% are novel. The identified structural variants are of high-quality, with an estimated false discovery rate of 3.2%. The concatenated length of all the structural variants is approximately 13.2% of the human reference genome. We annotate 1,929 loss-of-function structural variants affecting the coding sequence of 1,681 genes. We discover rare deletions in *HBA1*/*HBA2/HBB* associated with anemia. Furthermore, we identify structural variants related to immunity which differentiate the northern and southern Chinese populations. Our study describes the landscape of structural variants in the Chinese population and their contribution to phenotypes and disease.

## Abstract (web summary)

Although many studies have characterized genetic variation in human populations, few have investigated structural variation and few have been in non-European populations. Here, the authors have performed long read sequencing on 405 Chinese individuals to identify structural variants and link them to phenotypes.

## Introduction

Human genetic variants, comprising single-nucleotide variants (SNVs), small insertions or deletions (InDels), and structural variants (SVs), contribute to many physical traits and human diseases. SVs are defined as genomic rearrangements that range in length from 50 basepairs (bp) to megabases (Mb) or longer, and include different forms, such as deletion (DEL), insertion (INS), duplication (DUP), and inversion (INV)^1^. Accumulating evidence has demonstrated that SVs are associated with many human diseases, such as neurodevelopmental diseases and cancer^2–6^.

While substantial progress has been made in identifying SNVs and InDels based on short-read sequencing (SRS) technologies, the discovery and genotyping of SVs have been hampered due to the limited power of SRS to detect SVs that occur in repetitive regions with complex structures, which are common^7^. In addition, the current human reference genome includes a comprehensive map of SNVs and InDels depicted by SRS technologies^8,9^. More recently, third-generation sequencing (TGS) platforms such as Pacific Biosciences (PacBio) and Oxford Nanopore Technologies (ONT) have enabled long-read sequencing (LRS), which improves the sensitivity for SV discovery and contributes to improving the understanding of the SV spectrum in human genomes^10–14^.

Recently, a milestone study generated 15 human genomes using LRS with PacBio technology^11^. Despite the small sample size, the authors discovered 99,604 nonredundant SVs and 2238 SVs were shared by all 15 genomes. More recently, a population-scale SV study using LRS was reported in an Icelandic population^10^. The authors identified a median of 22,636 SVs in each sample, some of which might be Icelander specific. Icelanders are a North Germanic ethnic group that is historically coherent, with an estimated population of close to 341 thousand people^15^. In contrast, Han Chinese is the largest ethnic group in the world, with a total population size of ~1439 million^16^, comprising ~18.5% of the human population. Although several recent studies have reported SVs in Chinese genomes based on LRS^17–20^, the complexity and diversity of genetic variation characterized by SVs in a large-scale Chinese population are unclear.

In this study, we genotyped SVs in the Chinese population by performing whole-genome LRS of 405 unrelated Chinese, with 68 phenotypic and clinical measurements. We detected 132,312 nonredundant SVs, among which 59,814 (45.2%) were novel. The identified SVs were of high-quality, and 1080 (94.6%) and 145 (94.2%) singleton SVs were validated by PacBio high-fidelity (HiFi) sequencing and PCR experiments, respectively. We annotated 1,929 loss-of-function SVs affecting the coding sequences of 1,681 genes and further uncovered the impacts of SVs on clinical phenotypes and population differentiation. We discovered rare deletions in *HBA1*/*HBA2/HBB* associated with anemia. Furthermore, we identified SVs related to human immunity to differentiate northern and southern Chinese populations. Our study reveals the landscape of SVs in the Chinese population and provides insights into their roles contributing to phenotypes, diseases, and population adaptation.

## Results

### Sequencing, SV discovery, and validation

**Fig. 1:**
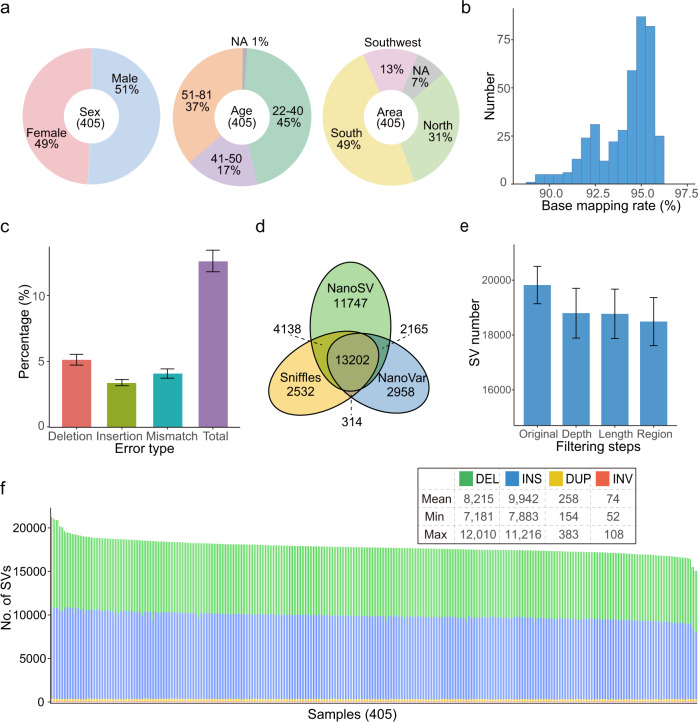
Overview of samples, datasets, and SVs. **a** Studied sample information overview, “NA” denotes not available. **b** Distribution of the base-mapping rate of clean data aligned to the reference genome GRCh38. **c** Error rate represented as percentage for each type (*n* = 405 per group). **d** Average number of SVs identified by Sniffles, NanoVar and NanoSV per individual and the overlap among them. **e** Average number of SVs after each filtering step (*n* = 405 per group), “Original” represents unfiltered SVs detected by at least two callers, “Depth”, “Length”, and “Region” represent SVs filtered according to the supported read number, extra-long intervals, and very low-complexity regions, respectively. **f** Number of SVs of each type in each individual. Data are represented as mean ± SD in **c** and **e**.

We performed whole-genome LRS of 405 unrelated Chinese individuals via the PromethION platform (ONT). Among all of the sequenced individuals, 206 (50.9%) were males and 199 (49.1%) were females. Their ages ranged from 22 to 81 years, with a median age of 42 years. While the ancestral regions of 30 individuals were unknown, the remaining 375 individuals came from 18 provinces in North (124 individuals), South (198), and Southwest (53) China (Fig. 1a and Supplementary Data 1). Among this study population, 68 phenotypic and clinical measurements from 327 individuals were obtained by health screening (Supplementary Data 2).

For the 405 Chinese individuals, a total of 20.7 terabases (Tb) of clean reads were obtained after quality control (Methods), with an average of 51.0 gigabases (Gb) per individual, representing an average depth of ~17 fold (Supplementary Fig. 1a and Supplementary Data 1). The clean reads were then mapped onto the human reference genome GRCh38, and the base-mapping rates for different individuals varied from 89.0 to 96.2%, with an average of 94.1% (Fig. 1b). The mean error rate of base mapping to the reference genome was 12.6%, ranging from 10.8 to 16.0% (Methods), which was similar to the rate reported in a recent study (median 11.6%)^10^. The percentages of deletions, insertions and substitutions (mismatches) were 5.1%, 3.4%, and 4.1%, respectively (Fig. 1c).

Four classes of canonical SVs (DEL, INS, DUP, and INV) with a length of at least 50 bp were detected. To obtain reliable SVs, we used three SV callers, Sniffles^21^, NanoVar^22^, and NanoSV^23^, all of which were specifically designed for SV detection from LRS (Supplementary Fig. 2a). We retained the SVs identified by at least two callers (Fig. 1d), which could effectively reduce the false-positive rate of SV detection, particularly for sequencing data with a lower depth (Supplementary Fig. 2b and Methods). In addition, we applied three filtering steps that removed an average of 1331 SVs per sample to further reduce unreliable SVs (Fig. 1e, Supplementary Data 3 and Methods). Finally, we identified 18,489 high-confidence SVs per sample on average, ranging from 15,439 to 22,505 (Fig. 1f and Supplementary Data 3). The numbers of SVs followed an approximately normal distribution (Supplementary Fig. 2c). DELs and INSs were predominant, and each sample contained an average of 8215 DELs (44.4%), 9942 INSs (53.8%), 258 DUPs (1.4%), and 74 INVs (0.4%) (Fig. 1f). A balanced number of DELs and INSs was also observed in previous LRS-based SV studies^10,11^, and the slightly higher ratio of INSs than DELs may be due to the DEL bias of GRCh38^24^.

We estimated the relationship between SV numbers and sequencing depth. The number of SVs increased only slightly when the depth was more than 15 fold (Supplementary Fig. 2b). The number of SVs was approximately 19,070 per sample at a 15-fold depth and increased to 20,378 at a 40-fold depth (Supplementary Fig. 2b). The limited increase in detected SV numbers beyond 15-fold depth using the methods that we employed indicated that 15-fold depth was adequate for the objectives of our study.

LRS technology such as ONT with high sequence error is more likely to lead to mismapping against the reference genome and therefore increase the false discovery of SVs^25^. To estimate the false discovery rate (FDR) of SVs using our SV identification strategy, SVs detected from a 15-fold ONT dataset (HG002, the child) were validated with a PacBio HiFi dataset from a parent-offspring trio in Genome in a Bottle (GIAB), whose base accuracy was up to 99.8%^26^. Among the 18,737 detected SVs in the HG002 dataset, the overall FDR was 3.2%, illustrating the reliability of SVs detected using our SV identification strategy from ONT reads with a 15-fold depth (Methods). INVs are generally enriched in false positives. To estimate the FDR of INVs, we further manually investigated the strand-specific alignment of long-read for the INV region using Integrative Genomics Viewer (IGV)^27^ (Supplementary Fig. 3a). We checked 85 INVs and detected four false positives with an estimated FDR of 4.7% (Supplementary Data 4).

### Comparison with published SV datasets

**Fig. 2:**
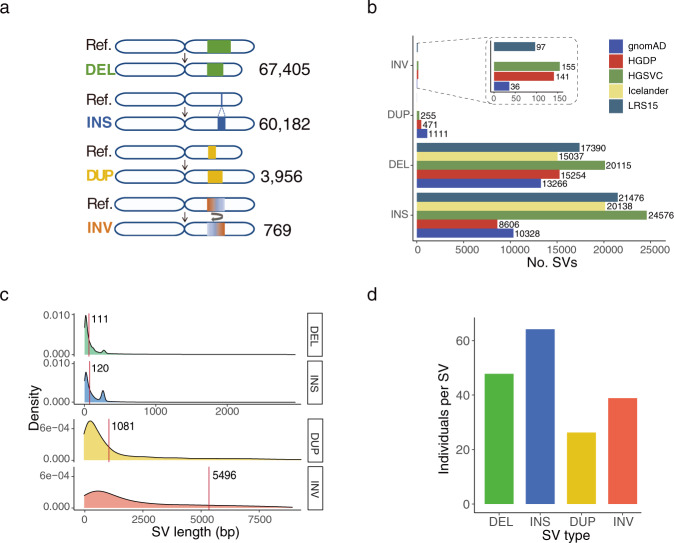
Properties of nonredundant SVs of each type. **a** Number of nonredundant SVs of all individuals. **b** Overlaps of SV number between our study and the previously published datasets for each SV type. Five published datasets, namely, LRS15^11^, gnomAD^8^, HGDP^9^, HGSVC^13, 14^, and Icelander^10^, were used in this study. The number on the bar graph indicates the actual number of SVs. **c** Length distribution for each SV type. The red line indicates the median length of each SV type. **d** The average number of individuals for merged nonredundant SVs of each type.

We merged the SVs detected from all the samples for each SV type and constructed a set of 132,312 nonredundant SVs, comprising 67,405 DELs, 60,182 INSs, 3956 DUPs, and 769 INVs (Fig. 2a). We compared our data with five previously published datasets generated using either SRS or LRS platforms. Our results showed greatest number of overlapping SVs (45,101) with HGSVC^13,14^, derived from multiple platforms and haplotype-resolved assemblies from multiple populations (Fig. 2b and Supplementary Fig. 4a, b). A total of 38,963 and 35,175 SVs overlapped with the datasets of LRS15^11^ and Icelanders^10^, respectively, which were generated using LRS platforms. The higher genetic diversity of HGSVC and LRS15 may contribute to the greater overlap with our dataset. There were 24,741 and 24,472 SVs that overlapped with the datasets of the Genome Aggregation Database (gnomAD)^8^ and the Human Genome Diversity Project (HGDP)^9^, which were generated by SRS platforms (Fig. 2b and Supplementary Fig. 4b). In total, 59,814 (45.2%) SVs identified based on our data have not been previously reported. We further examined the recovery of SVs in the previous datasets for each SV type. It was notable that although the total numbers of INSs and DELs were similar in our dataset, the recovered ratio of INSs to DELs for the LRS-based datasets was much higher than that for the SRS-based datasets (1.27 vs. 0.67), illustrating that LRS technology is particularly efficient in detecting INSs (Fig. 2b). Among our SVs that overlapped with the LRS and SRS datasets, SVs with relatively short lengths accounted for a high proportion (Supplementary Fig. 4c). The overlapping SVs from the SRS datasets tended to be longer than those from the LRS datasets, particularly for SVs longer than 2 kb. In addition, for SVs from Asian individuals in the published datasets, the recall rates were 59.9%, 49.9%, 28.7%, and 24.5% for HGSVC, LRS15, gnomAD, and HGDP, respectively (Supplementary Fig. 4d). The lower rates in gnomAD and HGDP may be due to the greater number of Asian individuals (~1310 and 416) and the higher genetic diversity in these datasets.

### Genomic features of SVs

The number of SVs generally increased at the ends of chromosome arms, particularly for DELs, INSs, and DUPs (Supplementary Fig. 5a). The subtelomeric bias of the long arms of chromosomes was higher than that of the short arms (Supplementary Fig. 5b-e), which was in accord with the pattern detected in an LRS-based SV study using a PacBio platform^11^.

We observed that the median lengths of INSs and DELs were 111 and 120 bp, respectively, which were significantly shorter than those of DUPs (1081 bp) and INVs (5496 bp, Fig. 2c). The longer lengths of DUPs and INVs were confirmed by our PacBio HiFi datasets as well as the PacBio HiFi datasets of the trio from GIAB, indicating that the observation was not specific to the ONT platform. The numbers of DELs and INSs rapidly decreased as their length increased. There were two clear peaks at sizes of ~300 bp and 6 kilobases (kb) for both DELs and INSs (Supplementary Fig. 6), corresponding to Alu elements and long interspersed nuclear elements (LINEs)^10,11^.

The concatenated length of all the SVs was 395.6 Mb, representing approximately 13.2% of the human reference genome, including 125.7 Mb of DELs, 19.8 Mb of INSs, 104.8 Mb of DUPs, and 145.2 Mb of INVs. On average, SVs affected 23.0 Mb (~0.8%) of the genome per individual, where the average lengths of DELs and INSs were 7.2 Mb (31.2% of the total SV length) and 3.7 Mb (15.9%), respectively. Despite their lower number, INVs (9.6 Mb, 41.7%) and DUPs (2.5 Mb, 11.1%) contributed equivalently to the total SV length due to their considerably longer lengths. Compared with the other SV types, INSs occurred more frequently in individuals, which may have been partly due to the DEL bias of GRCh38^12^ or the purification selection of functional INSs^8^ (Fig. 2d). A total of 75.4% of the SVs contained repeat sequences or interspersed elements (Supplementary Data 6), similar to the percentage of 78.3% detected in the Chinese Tibetan and Han populations^28^. This was consistent with the knowledge that SVs tend to occur in segments with more repetitive sequences^1^. Among all of these elements, VNTRs (25.1% of SVs) and SINEs (19.7%) were predominant (Supplementary Data 6).

### Allele frequencies of SVs

**Fig. 3:**
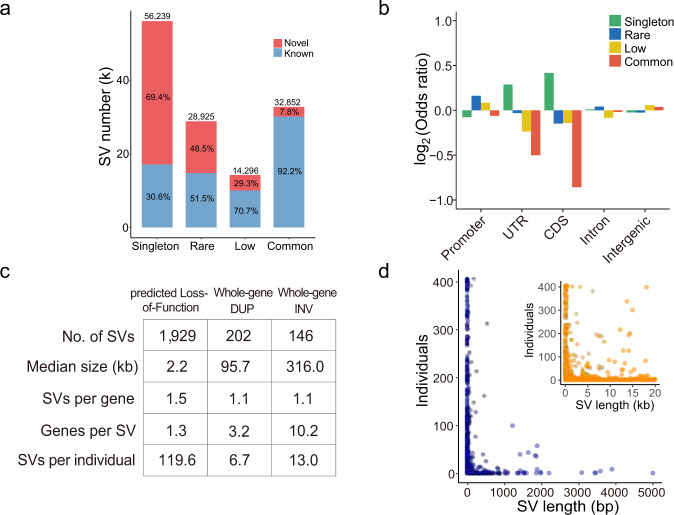
Allele frequency of SVs and functional annotation. **a** Number of known and novel SVs from each SV category based on the variant allele frequency (AF): singleton (allele count = 1), rare (allele count > 1 and AF ≤ 0.01), low (AF > 0.01 but ≤0.05), and common (AF > 0.05). **b** Enrichment analysis of the genomic location of SVs for each category. Two-sided Fisher’s exact test and Benjamini–Hochberg correction are applied for enrichment analysis. **c** Statistics of predicted loss-of-function (pLoF) SV, whole-gene DUP (WDUP), and whole-gene INV (WINV) SVs. **d** Individual number versus SV length for pLoF SVs. The blue figure shows SV lengths of 1–5000 bp, while the orange figure shows SV lengths of 0–20 kb.

Our datasets offer us an opportunity to identify SVs with a low frequency in a population. We grouped the SVs into four categories based on their allele frequencies (AF): singleton (allele count = 1), rare (allele count > 1 and AF ≤ 0.01), low (0.01 < AF ≤ 0.05) and common (AF > 0.05). Singletons (56,239) represented 42.5% of the total identified SVs (Fig. 3a). Additionally, there were 28,925 rare (21.9%), 14,296 low (10.8%), and 32,852 common (24.8%) SVs. Among the common SVs, 1264 (3.9%) were shared in all samples. The lower the AF values of identified SVs were, the larger the proportion of novel SVs that were not previously reported (Fig. 3a). Among all the 59,814 novel SVs, 65.2%, 23.5%, 7.0%, and 4.3% belonged to the singleton, rare, low and common frequency categories, respectively. Specifically, 69.4% of the singleton SVs were novel, similar to the percentage of novel singleton SNVs (72.8%) reported in Koreans^29^. In contrast, 7.8% of the common SVs were not previously reported (Fig. 3a, Supplementary Fig. 4e). The common SVs had higher allele frequency in Asians than in Africans (Mann–Whitney *U* test, *P* = 2.7 × 10^−7^) and Americans (Mann–Whitney *U* test, *P* = 0.029), as shown in the other SV datasets (Supplementary Fig. 4f).

Singleton SVs are prone to false-positive detection relative to other categories because they are detected in only one sample. To validate the accuracy of singletons, we designed primers for 154 randomly selected singleton DELs and INSs from 20 samples and validated 145 by PCR experiments (FDR = 5.8%) (Supplementary Data 7). In addition, we sequenced 10 samples using PacBio HiFi reads (average depth of 10.0-fold, Methods). We validated 1080 of our 1142 discovered singleton SVs using PacBio HiFi reads (FDR = 5.4%). We also validated 2618 of 2706 DUPs and INVs (FDR = 3.3%). In addition, we validated that 45 SVs that existed in more than two individuals by PCR experiments and the FDR was 4.5% (Supplementary Data 8).

To estimate the SV spectrum identified in the Chinese population in our study, we assessed SV numbers in different categories as the number of samples was increased through multiple sampling. A relatively stable number of common and low frequency SVs indicated that almost all the common and low frequency SVs in Chinese individuals could be identified in 405 of the samples included in this study (Supplementary Fig. 7). The continued increasing trends of the singleton and rare SVs suggested that a larger sample size was needed to sufficiently detect SVs with an AF ≤ 0.01 in Chinese individuals.

### Functional relevance of SVs

To explore their potential functions, we annotated SVs based on their genomic location, including coding sequences (CDSs), untranslated regions (UTRs), promoters and introns. A substantial percentage (37.6%) of the SVs were located in introns, while only 1.0%, 0.9%, and 1.7% of the SVs were located in promoters, UTRs, and CDSs, respectively (Table 1). Among all the SVs located in the UTRs and CDSs, singletons were significantly enriched compared with the other categories (*P* = 5.4 × 10^−4^ for singletons in UTRs and 5.9 × 10^−15^ for singletons in CDSs, Fisher’s exact test with Benjamini–Hochberg correction, Fig. 3b), suggesting that singleton SVs were more likely to have genetic functions.

**Table 1:** Gene annotation of SVs in each SV category.

Category	Promoter (%)	UTR (%)	CDS (%)	Intron (%)	Intergenic (%)	All
Singleton	542 (1.0)	594 (1.1)	1271 (2.3)	21,311 (37.9)	32,521 (57.8)	56,239
Rare	329 (1.1)	245 (0.8)	441 (1.5)	11,204 (38.7)	16,706 (57.8)	28,925
Low	154 (1.1)	105 (0.7)	219 (1.5)	5076 (35.5)	8742 (61.1)	14,296
Common	320 (1.0)	201 (0.6)	303 (0.9)	12,208 (37.2)	19,820 (60.3)	32,852
All	1345 (1.0)	1145 (0.9)	2234 (1.7)	49,799 (37.6)	77,789 (58.8)	132,312

Number and percentage of SVs affecting the promoters (1 kb upstream of gene), untranslated regions (UTRs), coding sequence regions (CDSs), and introns of protein-coding genes. SVs that did not intersect with genes were annotated as intergenic regions.

We further classified the SVs that interacted with the CDSs into three subgroups according to their breakpoint locations: predicted loss-of-function (pLoF), whole-gene duplication (WDUP), and whole-gene inversion (WINV) (Methods). While pLoF SVs are characterized by the deletion of coding nucleotides or alteration of open-reading frames, WDUPs generally cause the copy gain of an entire gene, and WINVs regulate gene expression by affecting the position and order of upstream enhancers and genes^8^. We annotated a total of 2277 SVs affecting the coding regions of 3176 distinct genes, including 1929 pLoF SVs, affecting the CDSs of 1681 genes, as well as 202 WDUPs and 146 WINVs, covering 581 and 1331 genes, respectively (Fig. 3c). Gene Ontology (GO) analysis revealed that 38 genes affected by pLoF SVs, which were significantly enriched in “immunoglobulin receptor binding” (odds ratio = 5.7, adjusted *P*-value = 7.2 × 10^−18^, Benjamini–Hochberg corrected, Supplementary Fig. 8).

On average, individuals carried 2.7 and 2.9 pLoF SVs from the singleton and rare categories, respectively. More than half of the pLoF SVs (57.6%) were singletons, and the median length of all pLoF SVs was 2.2 kb (Fig. 3d). The median length of common pLoF SVs was just 251 bp, while that of singleton pLoF SVs was up to 4480 bp (Supplementary Fig. 9). The structure and function of genes might be less tolerant to long pLoF SVs^8^; therefore, potential negative selection might reduce the frequency of these SVs to the rare or even singleton category.

### Phenotypic and clinical impacts of SVs

**Fig. 4:**
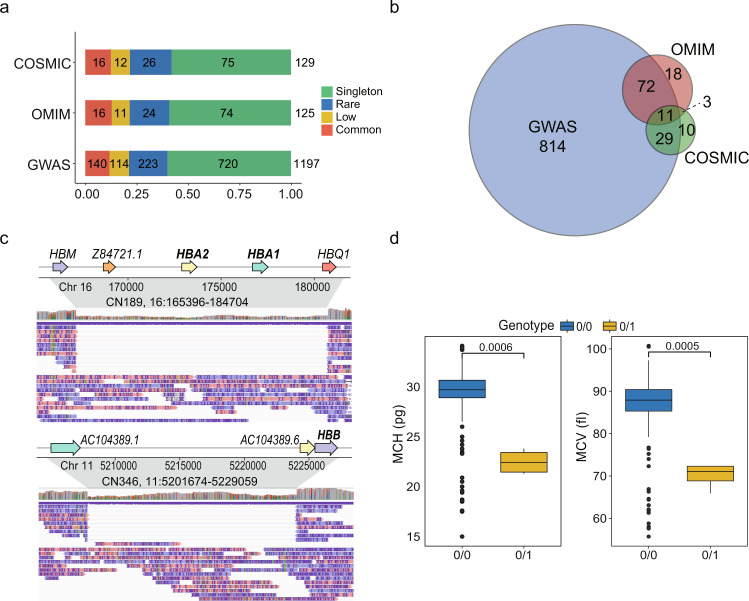
pLoF SVs associated with phenotypes and diseases. **a** Number of pLoF SVs with reported genes in GWAS, OMIM, and COSMIC for each SV category. **b** Number of genes associated with pLoF SVs. **c** Example of SVs affecting *HBB* and *HBA1*/*HBA2*, which are associated with anemia. Up: IGV screenshot of a 19.3 kb heterozygous DEL covering both *HBA1* and *HBA2*; bottom: IGV screenshot of a 27.4 kb heterozygous DEL covering *HBB*. **d** Mean corpuscular hemoglobin (MCH) and mean corpuscular volume (MCV) values of four individuals containing a 19.3 kb DEL (3 individuals) and a 27.4 kb DEL (1 individual) and the others (*n* = 323 for “0/0” and *n* = 4 for “0/1”). “0/0”: homozygous allele same as the reference; “0/1”: heterozygous variant. *P*-values were calculated using one-sided Student’s *t*-test. Boxplots show the different values between two genotypes. The center line in the box indicates the median, the lower and upper hinges indicate the first and third interquartile range (IQR). The lower and upper whiskers show the values greater than 25th quartile minus 1.5 × IQR and less than 75th quartile plus 1.5 × IQR, respectively. Where data beyond these ranges are shown as individual points.

To better understand how pLoF SVs impact clinical phenotypes and diseases, we annotated these SVs and their associated genes using the genome-wide association studies (GWAS) catalog^30^, online mendelian inheritance in man (OMIM) database^31^ and catalogue of somatic mutations in cancer (COSMIC)^32^. Among a total of 1929 pLoF SVs, 1231 (63.8%) intersected with genes cataloged in the above databases (Fig. 4a, Supplementary Data 9). Among the 1231 SVs, 58.1–60.2% belonged to the singleton category, which was consistent with our previous enrichment analysis showing that singletons were more likely to be functional (Fig. 3b). At the gene level, all 1929 pLoF SVs intersected with 1681 distinct genes, where 957 genes (56.9%) were annotated in the three databases (Fig. 4b and Supplementary Data 9).

Some phenotypically and clinically annotated SVs could be confirmed by our dataset. For example, we found a heterozygous rare DEL of 19.3 kb in three individuals, covering the genes hemoglobin subunit alpha 1 and 2 (*HBA1* and *HBA2*), whose dysfunctions are known to cause α-thalassemia^33^ (Fig. 4c). In addition, one individual exhibited a heterozygous DEL of 27.4 kb, containing gene hemoglobin subunit beta (*HBB*), whose dysfunction is known to cause serious hemoglobinopathies, such as sickle cell anemia and β-thalassemia^34^ (Fig. 4c). In this study, the average values of the mean corpuscular hemoglobin values (MCHs) and mean corpuscular volume values (MCVs) of individuals without two DELs were 39.4 ± 2.6 pg and 86.9 ± 6.3 fL, respectively. Interestingly, the MCHs (21.3–23.8 pg) and MCVs (65.9–72.3 fL) of these four individuals carrying the heterozygous DELs were significantly lower than those of individuals with the reference allele (*P* = 0.0006 and 0.0005 for MCH and MCV, respectively, *t*-test, Fig. 4d). In addition, we observed that there were 26 individuals with 19.3 kb DELs in gnomAD^8^, among which 24 (92.3%) individuals belonged to the East Asian population, suggesting that the DEL was population specific.

**Fig. 5:**
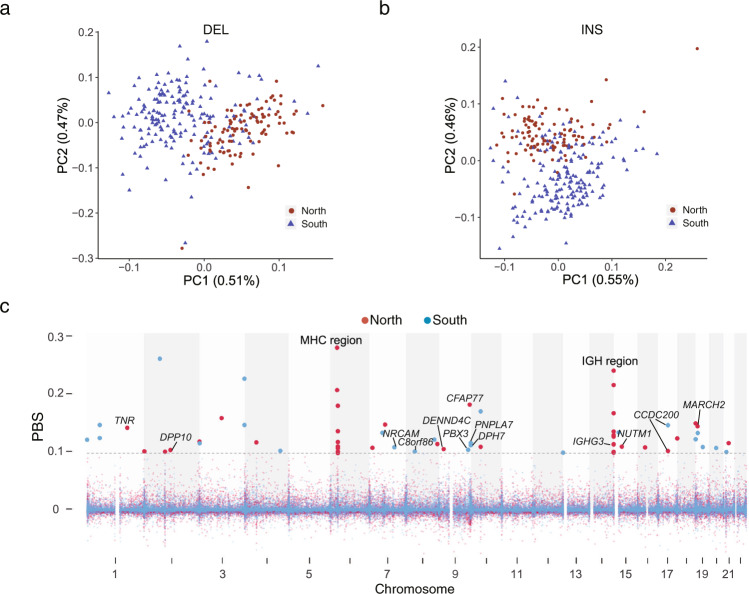
Genetic differentiation of SVs between subpopulations of Chinese individuals. **a** PCA of the two subpopulations: the northern and southern Chinese populations, based on DELs. The values in parentheses indicate the genetic variations explained by the first two PCs. **b** The first two PCs of the northern and southern Chinese populations based on INSs. **c** Population branch statistics (PBS) of the northern and southern Chinese populations; individuals from southeastern China regarded as an outgroup population. The gray line represents the 99.9% of the PBS ranked score for the northern and southern Chinese populations.

We further conducted GWAS of clinical phenotypes, such as biochemical, blood and urine compositions (Supplementary Data 2), based on the genotyped SVs with a minor allele frequency (MAF) > 0.05. We explored the population genetic properties between the northern and southern Chinese populations based on DELs and INSs. Principal component analysis (PCA) showed clear genetic diversity between the two groups, revealing that population structures were consistent with self-reported ancestry (Fig. 5a, b, Supplementary Fig. 10a-c). In addition, we calculated identity by state (IBS) for these individuals and found that the average IBS distances of individuals within South China, within North China and between South and North China were 0.786, 0.785, and 0.785, respectively, indicating little difference between subpopulations (Supplementary Fig. 10d). Therefore, we conducted GWAS by correcting for age, sex, BMI, and 10 PCs. The genomic inflation factor (λ_GC_) ranged from 1.00 to 1.05, with an average of 1.01, suggesting very little inflation. Finally, we found that 22 SVs on 14 chromosomes were significantly associated with 13 phenotypes (*P* < 1.7 × 10^−6^, the Bonferroni-corrected significance threshold, Supplementary Data 10). Among these SVs, five were located in introns, and the remaining 17 SVs were located in intergenic regions. For example, a 114 bp DEL with an MAF of 0.10 on chromosome 5 was significantly associated with urinary crystals (XTAL). This DEL is located in the intron of *SLC9A3* (sodium/hydrogen exchanger, isoform 3), which was previously found to be associated with ammonia metabolism, and regulates acidic or alkaline conditions in urine^35^ and thus affects calcium oxalate (CaOx) crystallization^36^.

### Population stratification based on SVs

Previous population genetics studies have shown the genetic differences between northern and southern Chinese individuals using SNP arrays and SRS-based WGS^17,37^. Herein, we computed the fixation index (*F*_ST_)-based population branch statistics (PBS) of the northern and southern Chinese populations. We observed 24 and 35 independent PBS signals across the genome in the northern and southern Chinese populations, respectively (Fig. 5c and Supplementary Data 11). Among these signals, eight and nine signals intersected with genes in the northern and southern Chinese populations. In the northern Chinese population, the top two PBS signals were in the major histocompatibility complex (MHC) region (6p21.3-p22.1) and in the immunoglobulin heavy (IGH) cluster locus (14q32.33). In the MHC region, nine SVs with PBS signals were located in the intergenic regions of *HLA-G*, *HLA-A*, *HLA-DRA*, *HLA-DRB5*, *HLA-DRB*1, *HLA-DQA1*, and *HLA-DPA1*, which was consistent with the fact that MHC is a known site of extreme genetic diversity across the human population and the report that MHC is under selection in the East Asian population^38^ (Fig. 5c and Supplementary Data 11). Notably, there were 10 SVs with PBS signals located in IGH cluster loci, such as the immunoglobulin heavy constant gamma 3 (*IGHG3*) and intergenic regions of *TEDC1*, *TMEM121*, *IGHA2*, *IGHE*, *IGHG2*, *IGHA1*, *IGHG1*, and *IGHG3* (Fig. 5c and Supplementary Data 11). Some SVs were in strong linkage disequilibrium (LD), such as the INV and subsequent DELs, while no haplotype blockage was observed in this region (Supplementary Fig. 11), which illustrated the genetic diversity among individuals, suggesting that the accumulation and combination of different genotypes of IGH genes might be associated with immune adaptation to diverse environments. In the southern Chinese population, we also detected PBS signals in the MHC and IGH regions. This result suggested that SVs in immunity-associated regions might have arisen due to long-term exposure to diverse environments and that different genes or alleles may be under selection for subpopulations of Chinese individuals to adapt to different habitats. In addition, a PBS signal with a 1.4 kb INS was located in the 13th intron of patatin like phospholipase domain containing 7 (*PNPLA7*). A previous study reported that *PNPLA7* was associated with hypertension based on RNA sequencing analysis^39^. Blood pressure and the prevalence of high blood pressure are greater in the northern than the southern Chinese population^40^. However, more evidence is needed to confirm that INS in intron of *PNPLA7* can cause changes in gene expression, leading to variations in blood pressure.

## Discussion

This study presented one of the largest LRS-based genomic datasets reported for Chinese population to date. We generated reliable reference sets of SVs and identified an average of 18,489 high-confidence SVs. Our SV dataset was much larger than those from SRS-based studies, in which an average of 4442 and 8202 SVs per human genome were detected^8,41^. Among the 132,312 nonredundant SVs described here, 45.2% were previously unreported. The large number of novel SVs identified in our study might be due to (1) the methodological improvement of detecting SVs using LRS technology instead of SRS technology^7,42^; (2) the large number of samples included in our study; and (3) the inclusion of the Chinese population, which has been poorly represented in previous studies.

LRS technology such as ONT with relatively high sequence error rates more likely to lead to mismapping against the reference genome and may cause unreliable SV detection. We used a multialgorithm ensemble approach and a stringent filtering strategy to improve SV detection. In addition, we performed orthogonal validation using PacBio HiFi sequencing and PCR experiments. The overall FDR of all the SVs was ~3.2% and that of singleton SVs was ~6%. Compared to a prior study in which an average of 22,755 SVs per genome were detected with an SV caller, SMRT-SV^11^, our study detected a lower number of SVs per genome (18,489). Our identification strategy reduced the false-positive rate, but it might also have decreased true positives. To estimate the false positives and true positives, we used the HG002 dataset from GIAB which provides a benchmark set generated from multiple platforms. We applied our SV calling strategy to HG002 and obtained 18,737 SVs, similar to the average number of our samples (18,489). If we only used one caller, we could obtain 21,153, 21,348, and 29,215 SVs from Sniffles, NanoSV, and NanoVar, respectively. Our identification strategy filtered out 2416, 2611, and 10,478 SVs from the three callers, among which only 171 (7.1%), 309 (11.8%), and 1,473 (14.1%), respectively, overlapped with the benchmark set in GIAB (Supplementary Data 5). In summary, among the 15,505 SVs that we filtered out, 1953 SVs (12.6%) overlapped with the benchmark set, which provided an estimate of the true positives that we missed. On the other hand, 13,552 of the SVs (87.4%) that we filtered out were not confirmed by the benchmark set. Therefore, it is challenging to balance precision and sensitivity in calling SVs from the human genome.

Our datasets enable us to explore SVs with a low frequency in the population. We provided several lines of evidence that singleton and rare SVs were more likely to be functional. In particular, pLoF SVs that altered coding regions and affected clinical phenotypes could be rare or even singletons in the population, as observed for the long DELs fully covering the *HBA1*/*HBA2* and *HBB* genes. Indeed, a recent study identified rare LoF variants in 26 genes through whole–exome sequencing, which were significantly associated with phenotypes^43^.

Base on abundant phenotypic and clinical measurements, our study presents a step for establishing a regional reference genome and provides a prospect for improving the interpretation of clinical genetics in Chinese population. To date, many diseases, including rare diseases and neurodevelopment-related diseases, have been verified to be caused by SVs^44,45^. However, it is still difficult to identify pathogenic SVs, even when conducting a whole-genome scan using LRS approaches, as the effects of SVs remain largely unknown, particularly for those in noncoding regions. Our SV dataset constructed from a large Chinese population could aid future LRS-based genomic studies in the pruning of candidate pathogenic variants.

In summary, given the substantially large number of unidentified SVs in the current reference human genome and population genomics data, this study represents an important effort to fill this knowledge gap and provides us with the opportunity to detect novel SVs associated with phenotypes, diseases and evolution.

## Methods

### Sample information

A total of 405 individuals were enrolled in this study (206 males and 199 females) with age varying from 22 to 81 years old (Fig. 1a). These individuals came from 18 provinces in China according to their self-reported original province. Northern and southern Chinese populations were distinguished based on Qinling Mountain-Huaihe River Line. Among them, 329 individuals were recruited at the Health Service Center of Sun Yat-sen University Cancer Center, where 68 clinical phenotypes from 327 (out of a total of 405 individuals) were collected. Additional 76 individuals from the West China Hospital of Sichuan University were included in this study. Written informed consent was obtained for all the individuals.

### Phenotype collection

Anthropometric measurements, including height, weight and body mass index (BMI), were obtained from an automatic electronic meter (SECA GM-1000, Seoul, Korea). Blood tests were processed by a hematology automated analyzer (SYSMEX XE-2100, Kobe, Japan). Urine tests were determined using an automated urine chemistry analyzer (ARKRAY4030, Tokyo, Japan) and a urinary tract infection analyzer (SYSMEX UF-1000i, Kobe, Japan). Biochemical detection was performed by an automatic modular analyzer (Cobas C701, Basel, Switzerland). Tumor markers such as alpha fetoprotein (AFP) and carcinoembryonic antigen (CEA) were measured by an immunology modular analyzer (Cobas 8000 e602, Basel, Switzerland).

### Library construction and long-read sequencing

High molecular weight genomic DNA of each individual was extracted from peripheral blood leukocytes using HiPure Tissue & Blood DNA Kit (D3018-03, Angen). For the Nanopore sequencing, DNA repair, end repair and adapter ligation were conducted during library preparation, and 2 µg DNA was Fragmented by g-TUBE (Covaris). DNA repair was performed using NEBNext FFPE DNA Repair Mix (M6630L, NEB). End repair was performed using NEBNext Ultra II End Repair/dA-Tailing Module (E7546L, NEB). Adapter ligation was performed using NEBNext Blunt/TA Ligase Master Mix (M0367L NEB) and Ligation Sequencing Kit 1D (SQK-LSK109, Oxford Nanopore Technologies). Library fragments with size of around 8 kb and 16 kb were selected for 329 individuals from Sun Yat-sen University Cancer Center and 76 individuals from Sichuan University, respectively. DNA was purified between each step using Agencourt AMPure XP beads (A63882, Beckman Coulter). DNA was quantified via a Qubit Fluorometer 2.0 (ThermoFisher Scientific, Waltham, MA). We carried out all long-read sequencing using the PromethION sequencer and 1D flow cell with protein pore R9.4.1 1D chemistry according to the manufacturer’s instructions. Reads were base-called in batches by guppy v3.2.8 using the default parameters during sequencing.

For the PacBio sequencing, the integrity of the DNA was determined with the Agilent 4200 Bioanalyzer (Agilent Technologies, Palo Alto, California). Eight micrograms of genomic DNA were sheared using g-Tubes (Covaris), and concentrated with AMPure PB magnetic beads. Each SMRT bell library was constructed using the Pacific Biosciences SMRT bell template prep kit 1.0. The constructed library was size-selected by Sage ELF for molecules 11–15 kb, followed by primer annealing and the binding of SMRT bell templates to polymerases with the DNA Polymerase Binding Kit (Pacific Bioscience). Sequencing was carried out on the Pacific Bioscience Sequel II platform for 30 h.

### Read quality control and mapping

In order to obtain sequences with high-quality, we detected base of quality at two ends of reads by NanoQC^46^ v0.8.1 and trimmed 30 bases of start and 20 bases of end for each raw read using NanoFilt^46^ v2.2.0 due to their lower quality (phred score < 7)^47^. We kept the reads with length longer than 500 bp and mean phred score higher than seven for downstream analysis. The statistics of length and quality value of cleaned reads was performed using NanoPlot^46^ v1.20.0. The cleaned reads were then aligned to the primary assembly of human reference genome GRCh38 using minimap2^48^ v2.15-r905 with the recommended option for ONT reads (-x map-ont) and the additional parameters “--MD -a”. Aligned files with SAM format were converted to BAM format and then sorted using SAMtools^49^ v1.9. Summary of aligned information for BAM file was conducted by command “stat” of SAMtools and region depth of aligned file was estimated by mosdepth^50^ v0.2.5. The read base-mapping rate and sequencing error rate were estimated with the method in Sequencing statistics of Supplementary Methods by Beyter, D. et al.^10^. Specifically, we aligned the cleaned reads to GRCh38 and estimated the rates of substitutions, insertions and deletions based on the mapping result, excluding secondary alignments and the soft-clipped sequences.

### Detection of high-confidence SVs

To obtain high-confidence SVs, we employed multiple tools to call SVs and conducted a series of filtering steps (Supplementary Fig. 2a). Sniffles^21^ v1.0.10, NanoVar^22^ v1.3.6 and NanoSV^23^ v1.2.4, which were SV callers specifically designed for long-read, were used to detect SVs. Sniffles was used with parameters “--min_support 2 --min_length 50 --num_reads_report -1 --min_seq_size 500 --genotype --report_BND --report_seq”. We set the following parameters for NanoVar: “--data_type ont --mincov 2 --minlen 50”. NanoSV had been run with the default parameters.

We merged the SV callsets of each individual derived from the above three SV callers for each type SV. We applied the Cluster Affinity Search Technique algorithm (CAST)^10,51^ to merge SVs independently for each SV type based on the variant position and length^10^. In order to facilitate the implementation of this algorithm for INSs, the end coordinate of INS was set as the sum of start coordinate and the SV length. First, we segregated all discovered SVs into nonoverlapping groups. For each group, we represented SVs as nodes in a graph and drew an edge between two SVs if they had a minimum mutual overlap of at least 50% of the length. The SV merging can be modeled by a corrupted clique graph. Consequently, we retained SVs detected by at least two callers for each individual. As suggested by benchmark analysis of LRS callers^52^, Sniffles showed the most balanced performance following by NanoVar and NanoSV for ONT reads. Therefore, we prioritized the results of Sniffles, followed by NanoVar. To obtain high-quality SVs, we then conducted three steps to filter out lower quality SVs (Supplementary Fig. 2a). First, we extracted SVs that were supported with at least three reads. A previous study used 1 Mb of threshold for discarding long DELs and INSs^10^. We checked the Database of Genomic Variants (DGV release 2020-02-25). We found that 99.97% of DELs and INSs were shorter than 2 Mb, and 99.08% INVs were shorter than 5 Mb. In addition, 99.77% INVs were shorter than 5 Mb in the HGSVC^13^. We therefore set 2 Mb threshold for DELs and INSs, 5 Mb for INVs and DUPs in our study. Then INSs and DELs with length larger than 2 Mb and INVs and DUPs larger than 5 Mb were discarded. Furthermore, sites in the centromere region with length of 61.9 Mb in 22 autosomes and the X chromosome were removed from further analysis. We identified and discarded SVs intersected with gap regions (marked as “N”) and high depth regions (≥500×, estimated by mosdepth^50^ v0.2.5) with BEDTools^53^ v2.27.1. The genomic position of gap and centromere regions were downloaded via the UCSC Table brower^54^.

To discern the relationship between the discovery rate of SVs and sequencing depth using different SV callers, we applied the above strategy to the Nanopore (ONT) reads of HG002 from Genome in a Bottle (GIAB). First, we obtained datasets with different depths ranging from 8× to 40× by randomly downsampling reads from the deep sequencing data of HG002. Then we detected SVs for each dataset with the same parameters using our strategy and thus produced SVs from the above three callers and the “Combine” methods. The method “Combine” means the SV shared by at least two of three callers (Supplementary Fig. 2b). The ratio threshold for SV supported reads was 0.2, which was equal to 3 supported reads for 15-fold data. The results combined from callers with higher sensitivity (NanoSV) and precision (NanoVar), and balanced performance (Sniffles) can be used for detecting high-confidence SVs. At the same time, our stringent strategy might discard some true SVs, which contributed to the smaller number of detected SVs compared with previously study^11^. In addition, the SV number became stable when the sequencing depth was more than 15×. For “Combine” method, 19,070 SVs were detected at depth of 15×, which was 93.6% of the total number (20,378) at depth of 40× (Supplementary Fig. 2b), indicating that the median depth (15×) sequencing data was a cost-effective method for genetic research at a population-scale.

### Nonredundant SVs and genotypes in the population

A large proportion of SVs were carried by multiple individuals because of their genetic similarity within population. To remove redundancies, we merged SVs of all the individuals using CAST algorithm. Followed a previous study, the most common SV in the population was used to represent the nonredundant SVs^10^. SVs were genotyped based on the variant allele balance (VAB). The genotype of individual was assigned as “0/0” if VAB ≤ 0.2, and genotype was “0/1” and “1/1” for 0.2 < VAB ≤ 0.8 and VAB > 0.8, respectively^55^. The threshold was same to prior study by Pedersen et al.^55^ and similar to that of vg toolkit (0.14 for default) when applying graph genotyping^56^.

After genotyping of SVs for all the individuals in the study, these SVs were classified with four categories based on the variant allele frequency (AF): singleton (allele count = 1), rare (allele count > 1 and AF ≤ 0.01), low (0.01 < AF ≤ 0.05) and common (AF > 0.05). To estimate the relationship between nonredundant SV number for different categories and sample size, we merged SVs randomly sampled from 100 to 405 samples while setting step size as 3, and repeated four times for each step. Then we calculated SV number for each category and regarded the average of four times as the estimate value of each step. We observed that the number of common SVs in population was relatively stable (Supplementary Fig. 7). As samples increased, the number of low SVs decreased and rare SVs increased, and the steps appeared when the sample number was a multiply of 50 due to the same integer threshold in this period. However, the total number of low and rare SVs increased with similar trend of singletons.

### False discovery rate (FDR) of detected SVs

In order to estimate the false discovery rate (FDR) for the detected SVs, we applied the strategy used in this study to the published dataset comprising of a parent-offspring trio with ONT and PacBio high-fidelity (HiFi) reads (accuracy > 99%). The depths of PacBio HiFi reads for HG002, HG003, and HG004 were 19.5×, 21.9×, and 21.6×, respectively. Simultaneously, we randomly selected 15.1× depth sequences from cleaned ONT reads for HG002 (child). After detecting SVs for each dataset via the same strategy as stated before, we compared SVs from HG002 with ONT reads to those of the trio with HiFi reads. For the 18,737 SVs detected by ONT reads, there were 1165 (6.2%) SVs not detected by HiFi reads of the trio. After manual investigation of IGV snapshot of HG002 with ONT reads and the corresponding PacBio HiFi reads, we finally found 608 false-positive SVs in HG002 with ONT reads, and the FDR of SV detection was 3.2%. Among them, false positives for DEL, INS, DUP, and INV were 459 (5.4%), 133 (1.3%), 11 (6.3%), and 4 (5.3%), respectively.

Singletons are known to have a higher error rate compared with the other categories because they existed in only one sample. To further orthogonally validate the accuracy of singletons uncovered in this study, we sequenced PacBio HiFi reads for 10 samples with depth ranging from 7.2 to 15.4 folds and then detected SVs applying above method. Among 1,142 singletons discovered by ONT reads for these samples, 62 SVs were false positive based on the validation of PacBio HiFi reads, with an FDR of 5.4% (https://github.com/xie-lab/PGC/tree/master/data). In addition, we investigated 743 INVs and 1963 DUPs in these samples. Among them, 707 INVs (95.2%) and 1911 DUPs (97.4%) were validated based on PacBio HiFi reads (FDR = 3.3%).

Besides the orthogonal validation for singletons using PacBio HiFi data, we further validated singletons using PCR experiments. We randomly selected singletons from 20 samples with SV lengths ranging from 60 to 810 bp (average of 293 bp) (Supplementary Data 7) and designed the primers using BatchPrimer3^57^ to amply the SV fragments. We conducted each PCR for positive sample, followed by negative sample and purified water without DNA, which were consider as negative control. Totally, we designed primers for randomly selected 154 DELs and INSs. Among them, amplified lengths for 145 (94.2%) primers were consistent with the targets, and nine primers failed to amplify target fragments (Supplementary Data 7). In addition, 45 primers were designed to validate the SVs in more than two individuals in this study (Supplementary Data 8). All PCR results was shown in https://github.com/xie-lab/PGC/tree/master/data.

### SV density of meta-chromosome

To compute the density of SVs of chromosomes, we normalized the lengths of all 22 autosomes and X chromosome. First, we split the chromosomes into p-arm and q-arm. The value of 0 to 1 corresponded to the telomere to centromere of p-arm, and the value of 1–2 corresponded to the centromere to telomere of q-arm. For each arm, we set a window of 100 kb and then calculated the SV number in each overlapping window. We normalized value of each window based on the positions relative to total length of each arm.

### Comparison of nonredundant SVs to the published datasets

To assess the known and novel SVs for our nonredundant SV call set, we compared it to some published datasets, including LRS study of 15 human genomes (LRS15)^11^, Human Genome Structural Variation Consortium (HGSVC)^13,14^, Icelander population^10^.Genome Aggregation Database (gnomAD v2.1)^8^ and Human Genome Diversity Project (HGDP)^9^, We extracted the position relative to GRCh38 and length information for each SV. The hg38 coordinates of gnomAD was converted by LifeOver (https://genome-store.ucsc.edu/)^58^ based on the original hg37 version. Copy number variation (CNV) with copy gain and copy loss were regarded as DUP and DEL, respectively. Additionally, the mobile element insertion (MEI) in those datasets were considered as INS. The end position of INS was defined as the sum of original end and the length of INS when comparing INSs between different datasets. We excluded INSs whose insertion length was not available because both SV length and position information should be taken into account. Intersected regions for each SV type between our study and the published datasets were conducted using BEDTools, and SVs were considered as overlapped if the reciprocal overlap was larger than 50%. In order to further detect the frequencies of novel common frequency SVs in different populations after comparing to the above five public datasets, we downloaded released LRS data from NCBI for 10, 18, and 13 individuals from Asia, Africa, and America, respectively, and then conducted the comparison again. The detail of sample information was in the code repertoire (https://github.com/xie-lab/PGC/tree/master/data).

### Repeat analysis of SV sequence

In order to better evaluate the pattern of repeat sequences for SVs, we selected the sequence of the individual with longest SV length in each merged SV. Consequently, we successfully obtained 55,476, 42,912, 3956, and 770 sequences for DEL, INS, DUP and INV, respectively (Supplementary Data 6). In aggregate, 103,114 (77.9% of total SVs) sequences were used for downstream analysis of repeat pattern. The repeat sequences were searched by RepeatMasker v4.0.9 (http://www.repeatmasker.org) based on databases of Dfam^59^ v3.0 and RepBase^60^ (release 10-26-2018) with command “RepeatMasker -species human -pa threads -gff -dir output sv_seq.fa” and Tandem Repeat Finder (TRF)^61^ v4.09 with command “trf 2 7 7 80 10 50 500 -f -d -m”. If the length of the repetitive sequence accounts for more than half of the total length of the SV, this SV was classified as the repeat family. For tandem repeats, the repeat unit length ≥ 7 bp were annotated as variable number of tandem repeats (VNTR). The VNTR regions for genome reference GRCh38 were downloaded via UCSC Table brower.

### Gene features of SVs

We annotated detected SVs based on the known protein-coding gene annotation file (gtf) corresponding GRCh38 from Ensembl release 95. We detected the intersection of SVs using BEDTools. Promoter was defined as the 1 kb region directly preceding the transcription start site of gene. We predicted Loss-of-Function (pLoF) SVs as follows: (1) DEL: overlap with at least one CDS; (2) INS: insertion directly into any CDS; (3) DUP and INV: partially overlap with at least one CDS^8^. In addition, INV and DUP were generally long, we hence defined DUP and INV that covered the whole-gene as WDUP and WINV, respectively. Although we did not consider WDUP and WINV as gene-disruptive SVs, we cannot rule out the possibility that they might enhance or regulate gene expression via duplication or cis-action. In addition, we labeled SVs as UTR-disruptive if at least one breakpoint was in 5’ or 3’ UTR and this SV was not intersected with CDS. Then we labeled SVs as promoter-disruptive if at least one breakpoint was in promoter of a gene and this SV was not intersected with CDS and UTR. We labeled SVs as intron-disruptive if both breakpoints were in same gene and this SV did not meet any of the above criteria. Ultimately, the remaining SVs that were not intersected with any protein-coding gene region (including promoter) was labeled as intergenic.

### Enrichment analysis of pLoF SVs and associated genes

For enrichment analysis of each gene feature annotation of SVs, the expected value was defined as the SV number in gene feature divided by the total number of SVs in population, and the SV number in certain category of this feature divided by the total number of SVs in this category was considered as observed value. The Fisher’s exact test was conducted in R^62^ v3.5.3 (http://www.R-project.org/). To assess functions and associated pathways of pLoF SVs, we performed enrichment analysis using GSEApy v0.9.16 (https://github.com/zqfang/GSEApy). The annotation files, including GO_Molecular_Function_2018, KEGG_2019_Human, GWAS_Catalog_2019, OMIM_Expanded, were downloaded from the Enrichr^63^ website (https://amp.pharm.mssm.edu/Enrichr). The *p*-value was calculated with Fisher’s exact test, and multiple testing of *p*-values were corrected by Benjamini–Hochberg method^64^.

### Population stratification and differentiation analysis

To assess the population stratification between northern or southern Chinese subpopulations, we performed principal component analysis (PCA) using EIGENSOFT^65^ v7.2.1. Previous study indicated that distinctly defined population structure can be uncovered by different type of SVs^9^. Therefore, 53,882 DELs and 54,605 INSs were independently used to estimate population stratification after filtering out those uniquely existing in southwest Chinese in this study. We calculated identity by state (IBS) to estimate kinship of individuals using PLINK^66^ v1.90b4. Then we calculated population branch statistics (PBS) for subpopulations using PBScan^67^ (v2020.03.16). All SVs that were polymorphic within subpopulations were used for further analysis. And we regarded the rank of 99.9% as the threshold for evidence of departure from neutrality. We calculated PBS using the northern, southern, and southeastern Chinese populations, and 42,299 SVs (MAF > 0.01) were used. The PBS thresholds for the northern and southern Chinese populations were 0.097. SVs with PBS score above the threshold within continuous 1 Mb were combined as an independent signal. Linkage disequilibrium (LD) analysis for SVs of PBS signals was conducted by Haploview^68^ v4.2.

### Genotype-phenotype association analysis

The 29,510 SVs with minor allele frequency (MAF) larger than 0.05 in 327 individuals with clinical phenotypes were used for the analysis. The genome-wide association study (GWAS) was performed using PLINK^66^ v1.90b4 with linear regression under an additive genetic model for the quantitative traits, and age, sex, body mass index (BMI), and the first ten principal components were included as covariates. When applying BMI GWAS, BMI itself was excluded from covariates. The association test for case-control was conducted using logistic regression module. The genome-wide significant threshold was set to be 1.7 × 10^−6^ through Bonferroni correction (0.05/29,510)^29^.

### Visualization of SVs with long-reads

Visualization of detected SVs was performed using Integrative Genomics Viewer (IGV)^27^ v2.8.6, which was specially updated for viewing variants of long-read. For target SVs, parameter “Link supplementary alignments” was selected to clearly identify heterozygous SVs based on the split reads. For INVs, the linked long reads with different colors (red and blue) indicated different strands when aligning to reference genome.

### Statistical analysis

The statistical tests used were described throughout the article and in the figures. The one-tailed Student’s *t*-test was performed to compare the clinical phenotype level between different genotypes of genes. We performed Benjamini–Hochberg correction for multiple comparisons using p.adjust in R. The enrichment analysis of singletons for different gene location was conducted by Fisher’s exact test. Benjamini–Hochberg corrected of *P*-value was used for multiple test analysis. All statistical tests were performed in R^62^ v3.5.3 (http://www.R-project.org/). In the boxplots, the upper and lower hinges represented the first and third quartile. The whiskers extended to the most extreme value within 1.5 times the interquartile range on either end of the distribution. The center line represented the median.

### Reporting summary

Further information on research design is available in the Nature Research Reporting Summary linked to this article.

## Supplementary information

Supplementary Information

Description of Additional Supplementary Files

Supplementary Data 1-11

Reporting Summary

## Data Availability

Our study is compliant with the “Guidance of the Ministry of Science and Technology (MOST) of China for the Review and Approval of Human Genetic Resources”. The summary data supporting the findings of this study are available either within the article and Supplementary Data files. The PacBio HiFi and PCR validations are available at https://github.com/xie-lab/PGC/tree/master/data. The VCF dataset has been deposited in the Genome Variation Map^69^ in National Genomics Data Center (NGDC)^70^, China National Center for Bioinformation (CNCB), under accession number GVM000132. The raw sequencing data have been deposited in the Genome Sequence Archive (GSA)^71^ in NGDC-CNCB under accession number HRA000792. The raw data are available under restricted access, which can be granted by the Data Access Committee (DAC). Access can be obtained by completing the application form via GSA. For detailed guidance on making the data access request, see GSA-Human_Request_Guide_for_Users [https://ngdc.cncb.ac.cn/gsa-human/document/GSA-Human_Request_Guide_for_Users_us.pdf]. The approximate response time for accession requests is about 10 working days. The public sequencing data and previously published SV callsets used in this study are listed at https://github.com/xie-lab/PGC/blob/master/data/ReleasedDataName.txt.

## Code availability

The codes of data analysis are publicly available at https://github.com/xie-lab/PGC (10.5281/zenodo.5520496^72^).
