# Supplementary material for: Structural variants in the Chinese population and their impact on phenotypes, diseases and population adaptation
Source: Nat Commun. 2021 Nov 11;12:6501. doi: 10.1038/s41467-021-26856-x (PMC8586011; doi:10.1038/s41467-021-26856-x)
Supplement: Supplementary file 1 — Supplementary Information [file 41467_2021_26856_MOESM1_ESM.pdf]

Structural variants in the Chinese population and their impact on phenotypes, diseases and population adaptation

Zhikun Wu, Zehang Jiang, Tong Li, Chuanbo Xie, Liansheng Zhao,
Jiaqi Yang, Shuai Ouyang, Yizhi Liu, Tao Li, Zhi Xie

Supplementary Figures

Contents

Supplementary Figure 1. Quality control of long-reads 1
Supplementary Figure 2. Workflow of SV calling and filtering 2
Supplementary Figure 3. Manual checking and statistics of INVs 3
Supplementary Figure 4. Statistics of SVs overlapped to other datasets 4
Supplementary Figure 5. SV distribution for DEL, INS, DUP and INV 6
Supplementary Figure 6. Length distribution for DELs and INSs 7
Supplementary Figure 7. The numbers of SVs with different categories as a function of number of samples used for detection 8
Supplementary Figure 8. GO enrichment analysis for pLoF SVs associated genes. 9
Supplementary Figure 9. Length distributions of pLoF SVs for different categories 10
Supplementary Figure 10. Principal components and kinship distribution of the northern and southern Chinese populations 11
Supplementary Figure 11. Population branch statistics (PBS) of SVs in IGH locus between the northern and southern Chinese populations 12

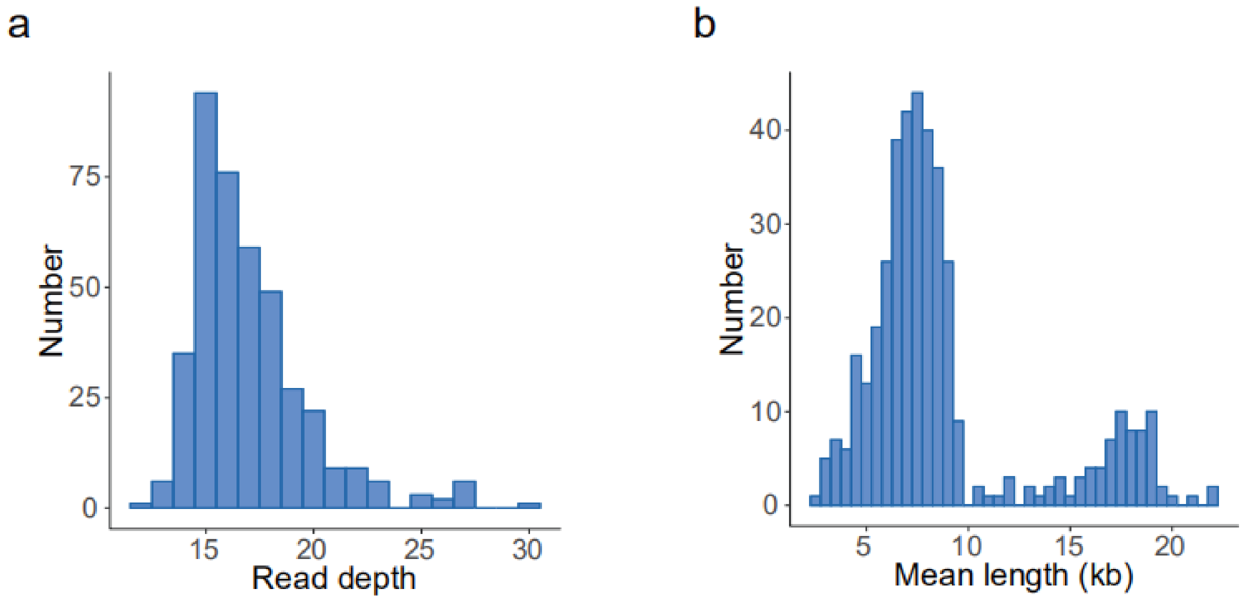

Supplementary Figure 1. Quality control of long-reads

a, Read depth distribution of clean data for 405 individuals.

b, Distribution of the average read length for 405 individuals.

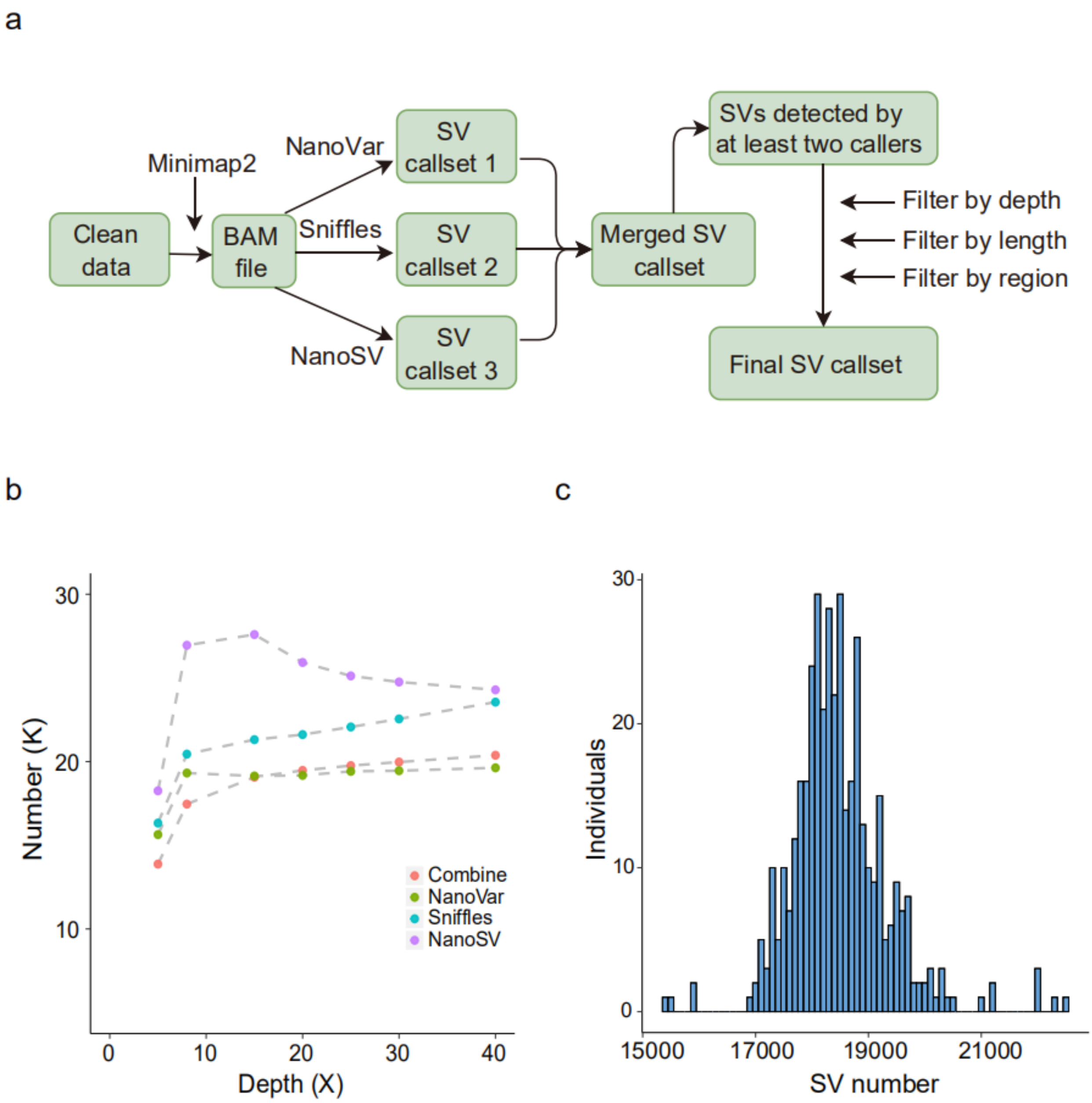

Supplementary Figure 2. Workflow of SV calling and filtering

a, Workflow of SV calling and filtering for each sample.

b, SV numbers of different callers for reads of different coverages, “Combine” means SVs detected by at least two callers. The threshold of read support of SVs is 0.2 of sequencing depth.

c, Distribution of final high-confidence SVs of all the individuals.

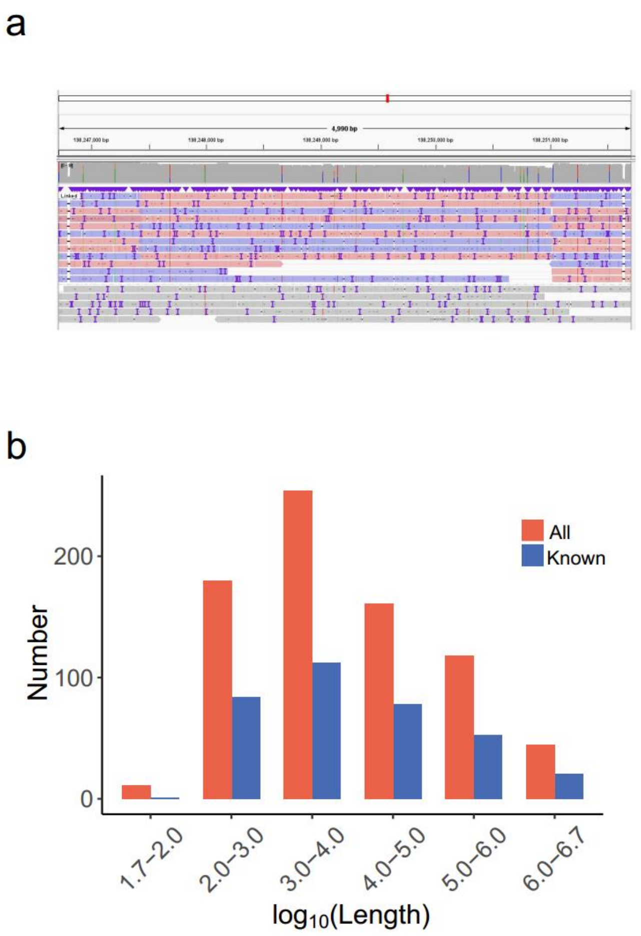

Supplementary Figure 3. Manual checking and statistics of INVs

a, An example of manual checking of INV (2:138247429-138251019). Snapshot of INV was conducted by IGV, and different colors (red and blue) indicate aligned different strands of the reference genome.

b, Length distribution of INVs in this study and those overlapped to the previous published datasets (LRS15, gnomAD, HGDP, HGSVC, InvFEST and nstd169). “All” indicates all INVs in our study, “Known” indicates overlapped INVs between our INVs and the above datasets.

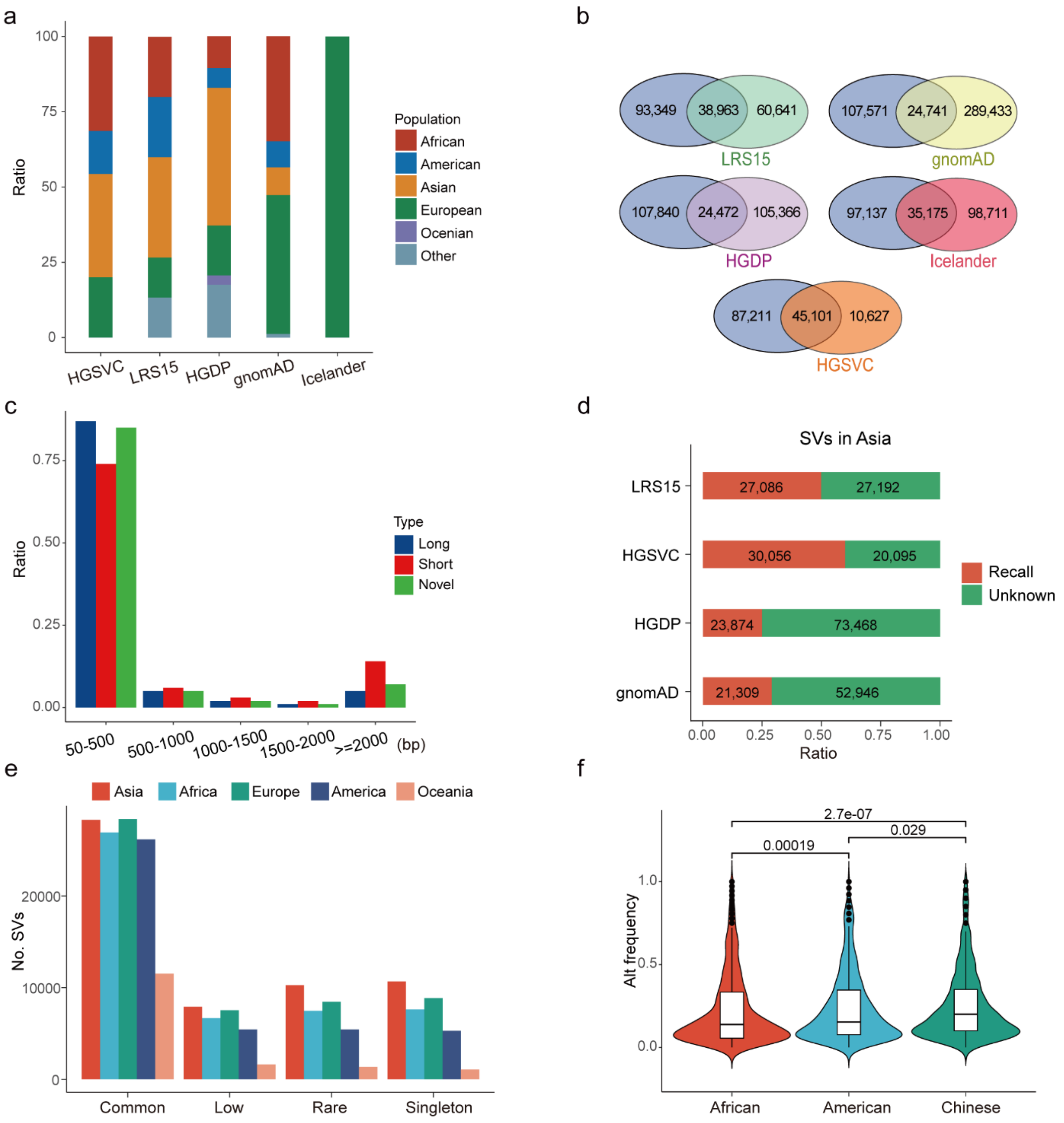

Supplementary Figure 4. Statistics of SVs overlapped to other datasets

a, Population frequencies of five published SV datasets used in this study.

b, The venn diagrams showing the overlapped and unique SVs between our SVs and the five published datasets.

c, Length distribution of SVs that were novel in our study and overlapped with datasets derived from long- or short-read sequencing platforms.

d, The recall rates of SVs that were detected from Asia.

e, The number of SVs overlapped to different populations in the five published datasets for each category.

f, Comparisons of the frequencies for the novel common frequency SVs overlapped to the released LRS datasets between African (n = 1,689), American (n = 1,772) and Chinese (n = 2,038). *P*-values were calculated using unadjusted two-sided Mann-Whitney *U* test. The violon plot shows the distribution of frequency levels and the inner box plot indicates the IQR (25th to 75th percentile, where center line indicating the median). The lower and upper whiskers show the values greater than 25th quartile minus 1.5 $\times$ IQR and less than 75th quartile plus 1.5 $\times$ IQR, respectively. Where data beyond these ranges are shown as individual points. The sample information was described in **Methods**.

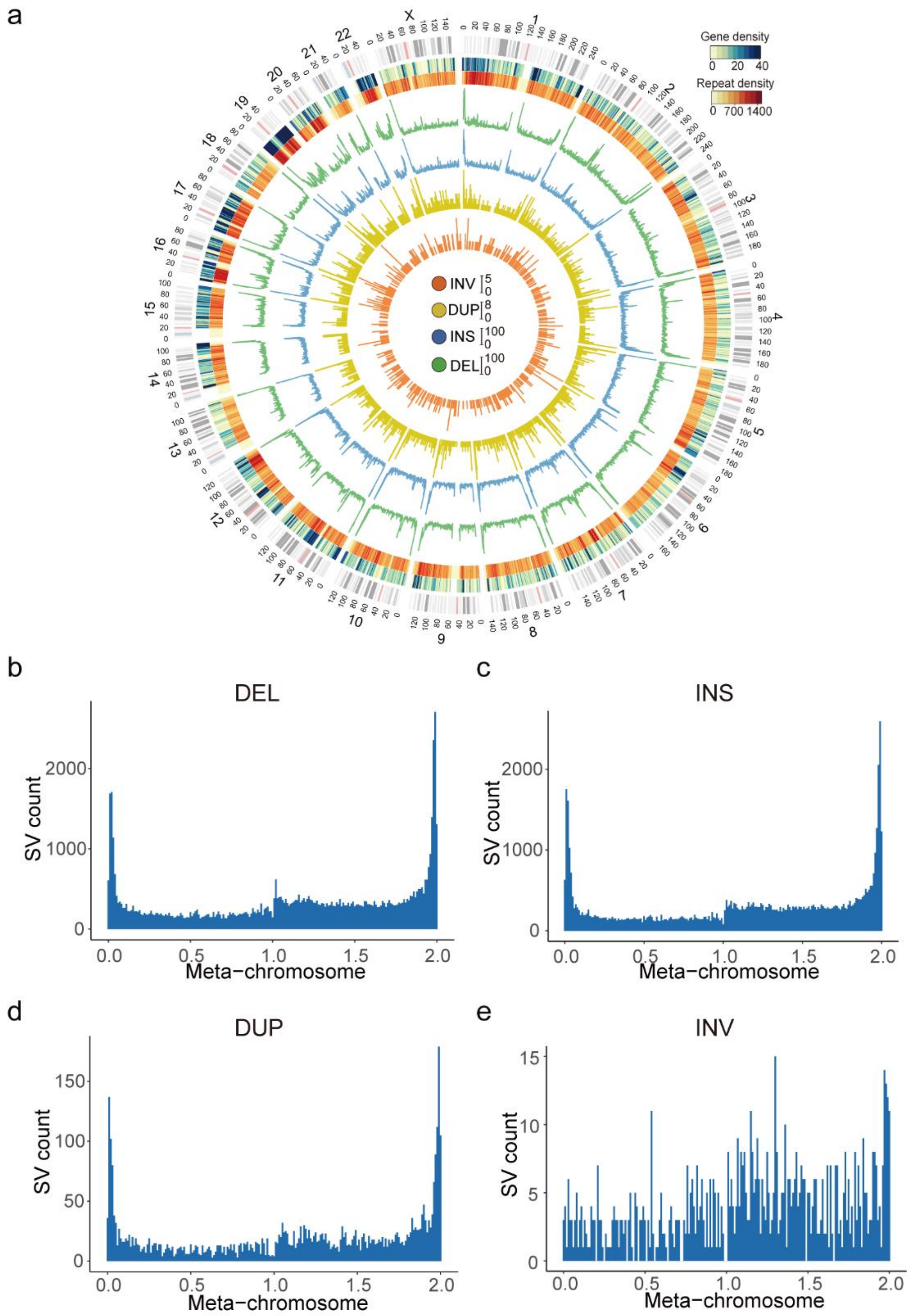

Supplementary Figure 5. SV distribution for DEL, INS, DUP and INV

a, Number of genes, repeats and SVs within 500 kb non-overlapping window across chromosomes. The two outer circles denote the distribution of genes and density of repeats, followed by distributions of DEL (green), INS (blue), DUP (yellow) and INV (orange).

b-e, SV distribution across meta-chromosome for DEL, INS, DUP and INV.

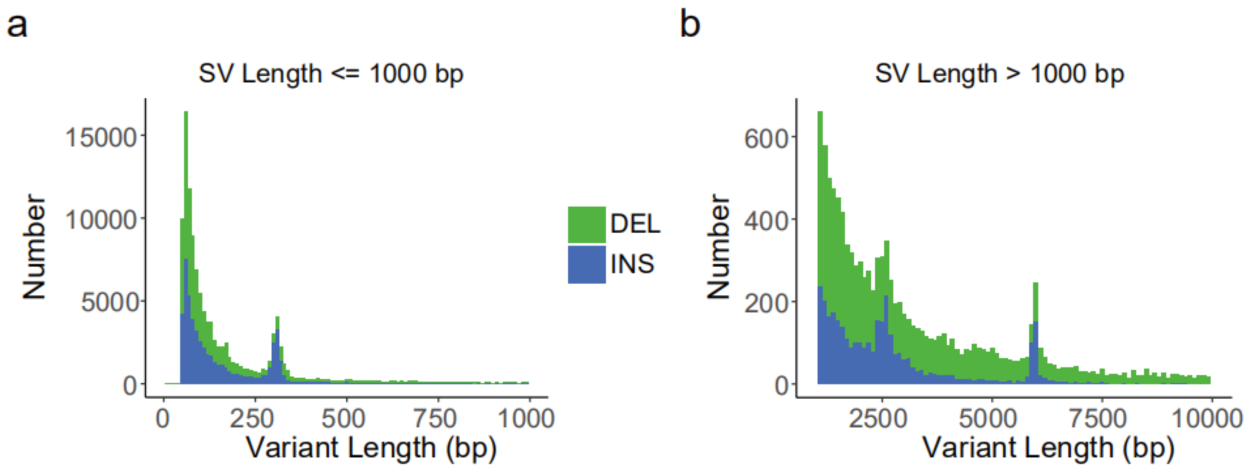

Supplementary Figure 6. Length distribution for DELs and INSs

SV distribution of DELs and INSs for (**a**) range of 50 bp to 1 kp and (**b**) range of 1 kb to 10 kb. Two noticeable peaks were observed at sizes around 300 bp and 6 kb.

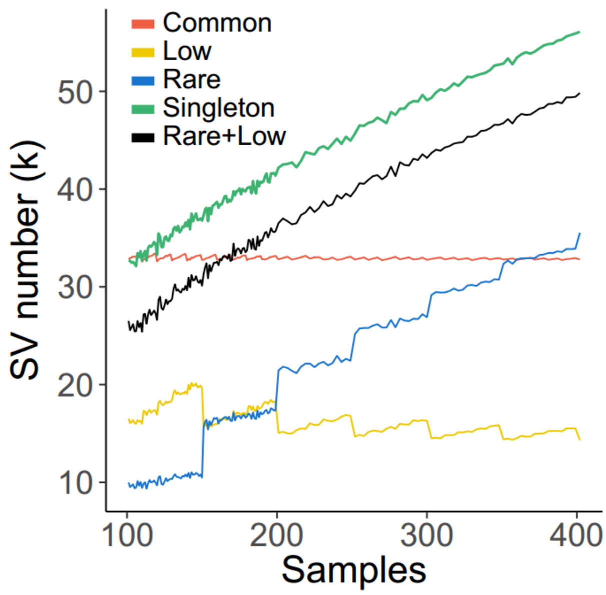

Supplementary Figure 7. The numbers of SVs with different categories as a function of number of samples used for detection

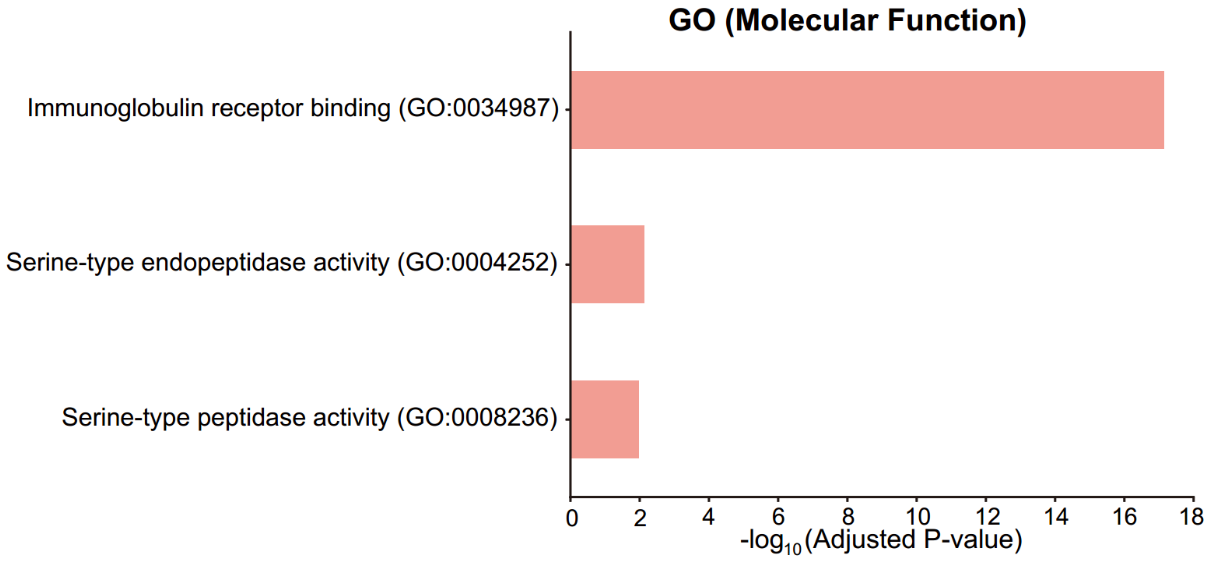

Supplementary Figure 8. GO enrichment analysis for pLoF SVs associated genes. *P*-values were calculated by two-sided Fisher's exact test which assumes a binomial distribution and independence for probability of any gene belonging to any set. Adjusted *P*-values were corrected by Benjamini-Hochberg method.

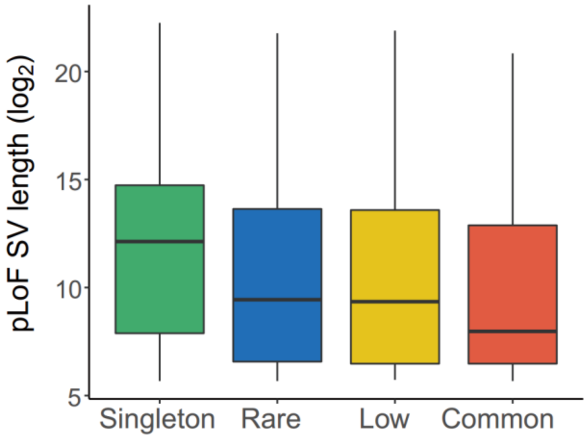

Supplementary Figure 9. Length distributions of pLoF SVs for different categories

Length distributions of pLoF SVs for four categories: singleton (n = 56,239), rare (n = 28,925), low (n = 14,296) and common (n = 32,852). Box plot indicates the IQR (25th to 75th percentile, where center line indicating the median). The lower and upper whiskers show the values greater than 25th quartile minus 1.5 × IQR and less than 75th quartile plus 1.5 × IQR, respectively.

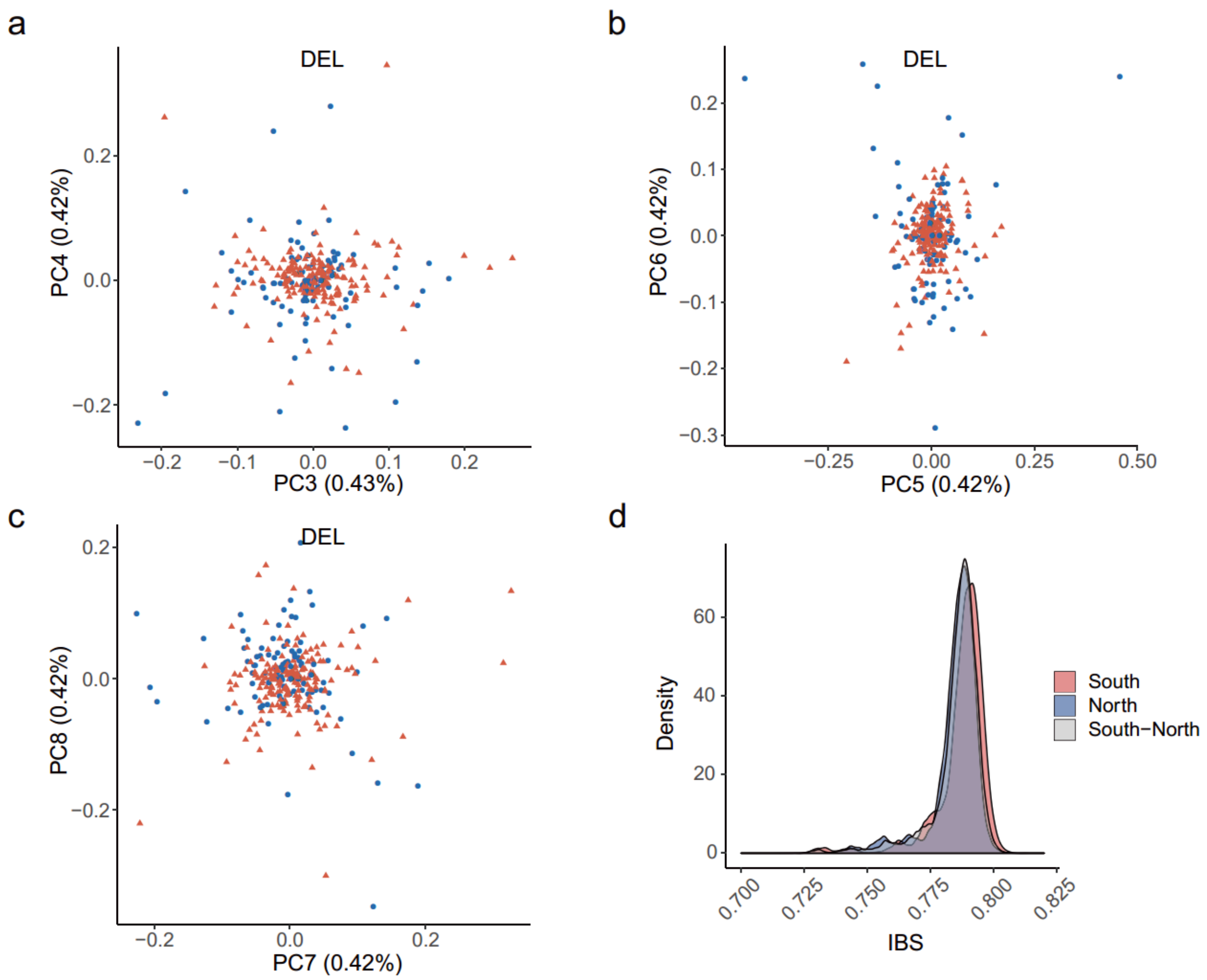

Supplementary Figure 10. Principal components and kinship distribution of the northern and southern Chinese populations

a, **b** and **c,** Principal components of the northern and southern Chinese populations based on DELs.

d, Identity by state (IBS) distribution between and within the northern and southern Chinese populations.

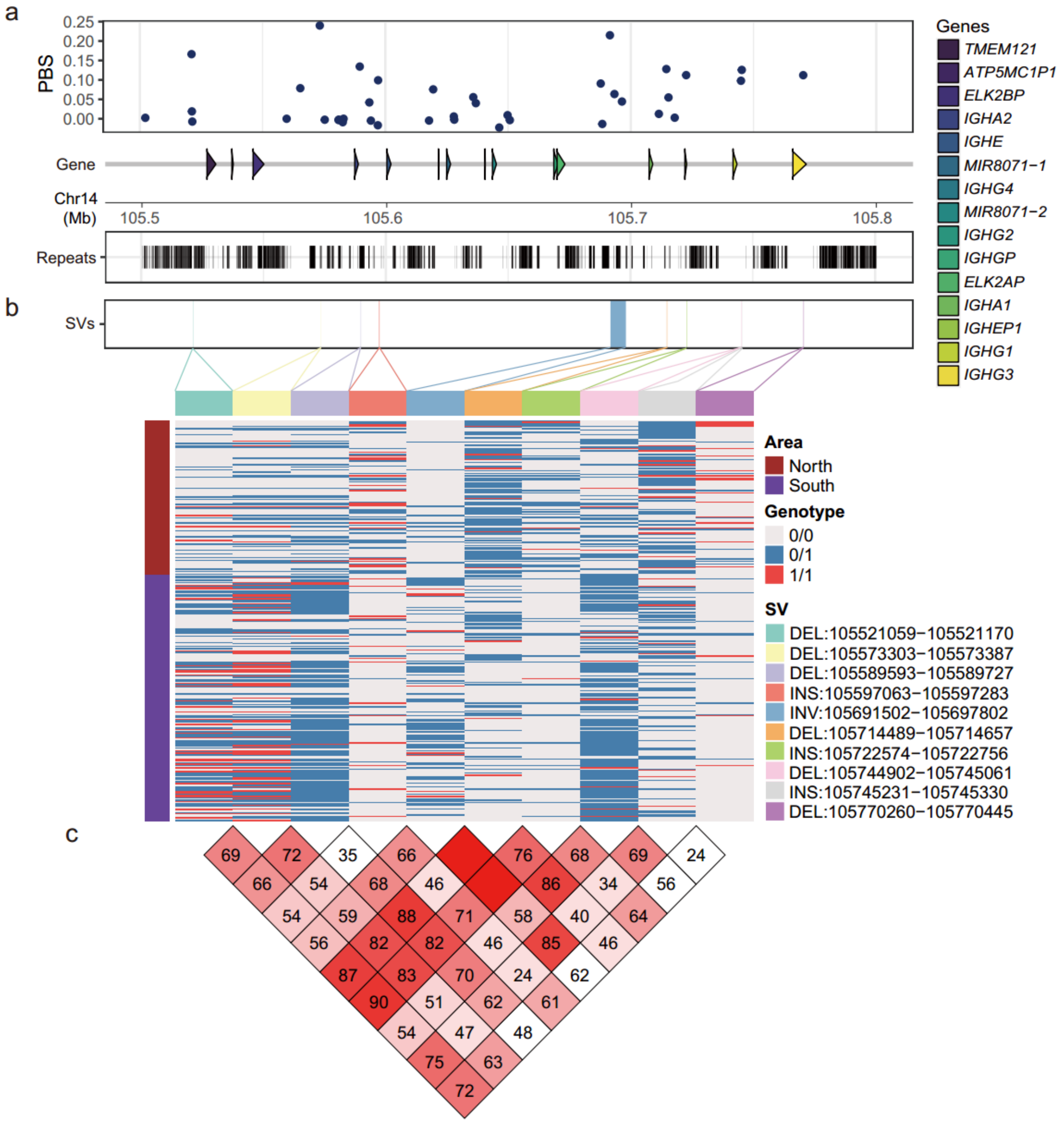

Supplementary Figure 11. Population branch statistics (PBS) of SVs in IGH locus between the northern and southern Chinese populations

a, PBS signals in IGH region of chromosome 14 for the northern Chinese population compared to the southern Chinese population.

b, SVs of PBS signals and genotype patterns between the two subpopulations.

c, Linkage disequilibrium (LD) analysis for SVs of PBS signals conducted by Haploview, the score indicates D’ of pair SVs.
