# Supplementary material for: Structural variants in the Chinese population and their impact on phenotypes, diseases and population adaptation
Source: Nat Commun. 2021 Nov 11;12:6501. doi: 10.1038/s41467-021-26856-x (PMC8586011; doi:10.1038/s41467-021-26856-x)
Supplement: Supplementary file 2 — Description of Additional Supplementary Files [file 41467_2021_26856_MOESM2_ESM.pdf]

Description of Additional Supplementary Files

File Name: Supplementary Data 1
Description: Summary of sample information and sequencing statistics

File Name: Supplementary Data 2
Description: . Summary of clinical traits

File Name: Supplementary Data 3
Description: SV statistics of different filtering processes for each sample

File Name: Supplementary Data 4
Description: Manually checked INVs of HG002

File Name: Supplementary Data 5
Description: Summary of Uniquely detected SVs by one caller and the overlaps with benchmark set

File Name: Supplementary Data 6
Description: Annotation of repeat sequences in SVs

File Name: Supplementary Data 7
Description: Summary of PCR validation results of singletons

File Name: Supplementary Data 8
Description: Summary of PCR validation results of novel non-singletons

File Name: Supplementary Data 9
Description: SVs and associated genes in GWAS, OMIM and COSMIC datasets

File Name: Supplementary Data 10
Description: Association for SVs and clinical phenotypes

File Name: Supplementary Data 11
Description: Information of SVs with significant PBS between Southern and Northern Chinese
