# Supplementary material for: Structural variants in the Chinese population and their impact on phenotypes, diseases and population adaptation
Source: Nat Commun. 2021 Nov 11;12:6501. doi: 10.1038/s41467-021-26856-x (PMC8586011; doi:10.1038/s41467-021-26856-x)
Supplement: Supplementary file 4 — Reporting Summary [file 41467_2021_26856_MOESM4_ESM.pdf]

nature portfolio

Corresponding author(s): Zhi Xie

Last updated by author(s): Sep 24, 2021

Reporting Summary

Nature Portfolio wishes to improve the reproducibility of the work that we publish. This form provides structure for consistency and transparency in reporting. For further information on Nature Portfolio policies, see our Editorial Policies and the Editorial Policy Checklist.

Statistics

For all statistical analyses, confirm that the following items are present in the figure legend, table legend, main text, or Methods section.

n/a	Confirmed	
[ ]	[x]	The exact sample size (*n*) for each experimental group/condition, given as a discrete number and unit of measurement
[x]	[ ]	A statement on whether measurements were taken from distinct samples or whether the same sample was measured repeatedly
[ ]	[x]	The statistical test(s) used AND whether they are one- or two-sided<br>*Only common tests should be described solely by name; describe more complex techniques in the Methods section.*
[x]	[ ]	A description of all covariates tested
[ ]	[x]	A description of any assumptions or corrections, such as tests of normality and adjustment for multiple comparisons
[ ]	[x]	A full description of the statistical parameters including central tendency (e.g. means) or other basic estimates (e.g. regression coefficient) AND variation (e.g. standard deviation) or associated estimates of uncertainty (e.g. confidence intervals)
[ ]	[x]	For null hypothesis testing, the test statistic (e.g. *F*, *t*, *r*) with confidence intervals, effect sizes, degrees of freedom and *P* value noted<br>*Give P values as exact values whenever suitable.*
[x]	[ ]	For Bayesian analysis, information on the choice of priors and Markov chain Monte Carlo settings
[x]	[ ]	For hierarchical and complex designs, identification of the appropriate level for tests and full reporting of outcomes
[ ]	[x]	Estimates of effect sizes (e.g. Cohen's *d*, Pearson's *r*), indicating how they were calculated

Our web collection on statistics for biologists contains articles on many of the points above.

Software and code

Policy information about availability of computer code

Data collection

Description of data collection and software used is available under sections Sample information, Phenotype collection, Library construction and long-read sequencing in Methods. In this study, 329 individuals were recruited at the Health Service Center of Sun Yat-sen University Cancer Center, where 68 clinical phenotypes from 327 were collected. Library fragments with size of around 8 kb and 16 kb were selected for 329 individuals from Sun Yat-sen University Cancer Center and 76 individuals from Sichuan University, respectively. We carried out all long-read sequencing using the PromethION sequencer and 1D flow cell with protein pore R9.4.1 1D chemistry according to the manufacturer's instructions. Reads were base-called in batches by guppy v3.2.8 using the default parameters during sequencing. PacBio HiFi data were carried out on the Pacific Bioscience Sequel II platform for 10 samples for orthogonal validation.

Data analysis

SOFTWARE	VERSION	REFERENCE	LINK
Guppy	v3.2.8	N/A	https://community.nanoporetech.com/protocols/Guppy-protocol/
NanoQC	v0.8.1	De Coster et al., 2018	https://github.com/wdecoster/nanoQC
NnaoFilt	v2.2.0	De Coster et al., 2018	https://github.com/wdecoster/nanofilt
NanoPlot	v1.20.0	De Coster et al., 2018	https://github.com/wdecoster/NanoPlot
minimap2	v2.15-r905	Li, 2018	https://github.com/lh3/minimap2
SAMtools	v1.9	Li et al., 2009	https://github.com/samtools/samtools
SeqKit	v0.10.1	Shen et al., 2016	https://github.com/shenwei356/seqkit
mosdepth	v0.2.5	Pedersen and Quinlan, 2018	https://github.com/brentp/mosdepth
Sniffles	v1.0.10	Sedlazeck et al., 2018	https://github.com/fritzsedlazeck/Sniffles
NanoVar	v1.3.6	Tham et al., 2020	https://github.com/benoukraflab/NanoVar
NanoSV	v1.2.4	Cretu Stancu et al., 2017	https://github.com/mroosmalen/nanosv
BEDTools	v2.27.1	Quinlan and Hall, 2010	https://bedtools.readthedocs.io/en/latest/
LifeOver	v377	Kuhn, R.M. et al., 2013	https://genome-store.ucsc.edu/

EIGENSOFT	v7.2.1	Price et al., 2006	https://github.com/DReichLab/EIG
RepeatMasker	v4.0.9	N/A	http://www.repeatmasker.org/
TRF	v4.09	Benson, G. 1999	https://tandem.bu.edu/trf/trf.html
Dfam	v3.0	Hubley et al., 2016	https://dfam.org/home
RepBase	N/A	Bao et al., 2015	https://www.girinst.org/repbase/
BatchPrimer3	v1.0	You, F.M. et al., 2008	https://wheat.pw.usda.gov/demos/BatchPrimer3/
GSEApy	v0.9.16	Zhuoqing Fang	https://github.com/zqfang/GSEApy
Enrichr	v2016	Kuleshov, M.V. et al.,	https://amp.pharm.mssm.edu/Enrichr
Integrative Genomics Viewer (IGV)	v2.8.6	Robinson et al., 2017	http://software.broadinstitute.org/software/igv/
PBScan	v2020.03.16	Hämälä, T. et al.,	https://github.com/thamala/PBScan
PLINK	v1.90b4	Purcell, S. et al., 2007	http://zzz.bwh.harvard.edu/plink/
Haploview	v4.2	Barrett, J.C., 2004	https://www.broadinstitute.org/haploview/haploview
R	v3.53	Team, T.R.C. 2020	http://www.R-project.org/

For manuscripts utilizing custom algorithms or software that are central to the research but not yet described in published literature, software must be made available to editors and reviewers. We strongly encourage code deposition in a community repository (e.g. GitHub). See the Nature Portfolio guidelines for submitting code & software for further information.

Data

Policy information about availability of data

All manuscripts must include a data availability statement. This statement should provide the following information, where applicable:

- Accession codes, unique identifiers, or web links for publicly available datasets
- A description of any restrictions on data availability
- For clinical datasets or third party data, please ensure that the statement adheres to our policy

Our study is compliant with the "Guidance of the Ministry of Science and Technology (MOST) of China for the Review and Approval of Human Genetic Resources". The PCR validations are available at https://github.com/xie-lab/PGC/tree/master/data. The VCF dataset has been deposited in the Genome Variation Map in National Genomics Data Center (NGDC), China National Center for Bioinformation (CNCB), under accession number GVM000132 (http://bigd.big.ac.cn/gvm/getProjectDetail?project=GVM000132). The raw sequence data have been deposited in the Genome Sequence Archive (GSA) in NGDC-CNCB under accession number HRA000792 (https://ngdc.cncb.ac.cn/gsa-human/browse/HRA000792). And the raw data are available under controlled access for granting by the corresponding Data Access Committee (DAC). The access need request by completing the application form via GSA. For detail guidance on making data access request, see https://ngdc.cncb.ac.cn/gsa-human/document/GSA-Human_Request_Guide_for_Users_us.pdf. The approximate response time for accession requests within 10 working days. The publicly available raw sequence data were downloaded from NCBI SRA under accession codes SRX8948932-SRX8948933, SRX9063411, SRX9596125-SRX9596129, SRX9596131-SRX9596135, SRX9847571, SRX9847600, SRX9847604, SRX9847780, SRX10073616-SRX10073618, SRX10073624, SRX10073710-SRX10073717, SRX10073721, SRX10247553-SRX10247556, SRX10247571-SRX10247572, SRX11061546-SRX11061551, ERX3990360, ERX3990354-ERX3990359, ERX3990366-ERX3990371, SRR13867040 and SRR13867065. HG002 long read ONT data were downloaded from https://ftp-trace.ncbi.nlm.nih.gov/ReferenceSamples/giab/data/AshkenazimTrio/HG002_NA24385_son/UCSC_Ultralong_OxfordNanopore_Promethion/. Other information of previously public SV callsets used in this study was at https://github.com/xie-lab/PGC/blob/master/data/ReleasedDataName.txt.

Field-specific reporting

Please select the one below that is the best fit for your research. If you are not sure, read the appropriate sections before making your selection.

☒ Life sciences ☐ Behavioural & social sciences ☐ Ecological, evolutionary & environmental sciences

For a reference copy of the document with all sections, see nature.com/documents/nr-reporting-summary-flat.pdf

Life sciences study design

All studies must disclose on these points even when the disclosure is negative.

Sample size	The sample size used in this study were not predetermined. The number of individuals is sufficient to detect high-confidence SVs for Chinese.
Data exclusions	Except for 30 individuals whose ancestral regions were unknown, 375 individuals were from 18 provinces in the North (124 individuals), South (198) and Southwest (53) of China. Among them, 68 phenotypic and clinical measurements for 327 individuals were obtained by health screening. Thus, phenotype data of these 327 individuals were used for gene-phenotype associations.
Replication	The raw data had been deposited in public repositories. The previously published data were also listed at https://github.com/xie-lab/PGC/blob/master/data/ReleasedDataName.txt. The codes of data analysis are publicly available at https://github.com/xie-lab/PGC, and the tools and corresponding version in this study were listed in “Data”. All these ensure the reproducibility of the experimental findings. We also performed orthogonal validation using PacBio high-fidelity (HiFi) sequencing and PCR experiments. And these validation data were available at https://github.com/xie-lab/PGC/blob/master/data.
Randomization	We randomly recruited health samples from the Health Service Center of Sun Yat-sen University Cancer Center and the West China Hospital of Sichuan University. In order to avoid bias of age/region, we stratified different age into younger group (20-45 years old) and elder group (>50 years old). In case of potential region bias, we balanced northern and southern sample size as much as possible.
Blinding	Blinding is not relevant to this study.

Reporting for specific materials, systems and methods

We require information from authors about some types of materials, experimental systems and methods used in many studies. Here, indicate whether each material, system or method listed is relevant to your study. If you are not sure if a list item applies to your research, read the appropriate section before selecting a response.

Materials & experimental systems

n/a	Involved in the study
☒	☐ Antibodies
☒	☐ Eukaryotic cell lines
☒	☐ Palaeontology and archaeology
☒	☐ Animals and other organisms
☐	☒ Human research participants
☒	☐ Clinical data
☒	☐ Dual use research of concern

Methods

n/a	Involved in the study
☒	☐ ChIP-seq
☒	☐ Flow cytometry
☒	☐ MRI-based neuroimaging

Human research participants

Policy information about studies involving human research participants

Population characteristics	A total of 405 healthy individuals who did not report any treatment history or not currently diagnosed as chronic diseases or cancer were enrolled in this study (206 males and 199 females) with age varying from 22 to 81 years old. These individuals came from 18 provinces in China according to their self-reported original province. Northern and southern Chinese were distinguished based on Qinling Mountain-Huaihe River Line. The genotypic information is unclear before recruitment until we got the genotypes based on SVs detected in our study.
Recruitment	329 individuals were recruited at the Health Service Center of Sun Yat-sen University Cancer Center, where 68 clinical phenotypes from 327 individuals were collected. Additional 76 individuals from the West China Hospital of Sichuan University were included in this study. These individuals came from 18 provinces in China according to their self-reported original province. Northern and southern Chinese were distinguished based on Qinling Mountain-Huaihe River Line. We found that PCA results were in consistent with self-reported province-origin. Even if some individuals were inaccurate, it may impact the result of PBS. And the affect is still small because the statistics of PBS mainly depends on the frequency of SVs of the sub-populations.
Ethics oversight	This research was approved by the Ethics Committee of ZhongShan Ophthalmic Center, Sun Yat-sen University (2019KYPJ111) and West China Hospital, Sichuan University (2018-120). Clinical data and biological specimens were obtained from the individuals with written informed consent.

Note that full information on the approval of the study protocol must also be provided in the manuscript.
